# Alternating Orthogonal Switching in a Thiophenyl‐Phenyl‐Bis‐Azobenzene Switch

**DOI:** 10.1002/chem.202501976

**Published:** 2025-08-22

**Authors:** Dazhong Sun, Nils Oberhof, Kai Hanke, Anne Kunz, Andreas H. Heindl, Lukas Kaltschnee, Christina M. Thiele, Chavdar Slavov, Andreas Dreuw, Josef Wachtveitl, Hermann A. Wegner

**Affiliations:** ^1^ Institute of Physical and Theoretical Chemistry Goethe University Max‐von‐Laue Str. 7 Frankfurt am Main Germany; ^2^ Interdisciplinary Center for Scientific Computing (IWR) University of Heidelberg Im Neuenheimer Feld 205 69120 Heidelberg Germany; ^3^ Institute of Organic Chemistry Justus Liebig University Giessen Heinrich‐Buff‐Ring 17 35392 Giessen Germany; ^4^ Center for Materials Research (ZfM/LaMa) Justus‐Liebig University Giessen Heinrich‐Buff‐Ring 16 35392 Giessen Germany; ^5^ Clemens‐Schöpf‐Institute for Organic Chemistry and Biochemistry Technical University of Darmstadt Peter‐Grünberg‐Str. 16 Darmstadt 64287 Germany; ^6^ Department of Chemistry University of South Florida 4202 E. Fowler Avenue Tampa 33620 USA

**Keywords:** azobenzene, computations, orthogonal switching, photochemistry, ultrafast dynamics

## Abstract

To design efficient molecular information storage systems with multi‐photoswitchable entities, orthogonal isomerization of the different switchable moieties is essential. Various challenges, like unintentional energy transfer, spectral overlap, and other energy dissipation channels, have to be addressed by intelligent molecule design. In this context, we took advantage of calculations to design a bis‐azobenzene switch, which consists of a phenyl‐ and a thiophenylazobenzene moiety in *meta*‐connection to reduce π‐conjugation. Ultrafast spectroscopy and computational studies confirmed that this bis‐photoswitch exhibits alternating orthogonal switching behavior when irradiated with light of different wavelengths. These results represent a significant advancement toward the development of efficient and adaptable organic multi‐photoswitches for applications, such as information storage, molecular machines, or smart materials.

## Graphical abstract

This study employs computational calculations and ultrafast spectroscopy to design and probe alternate isomerization in a bis‐azobenzene molecule. The phenyl‐ and thiophenylazobenzene moieties exhibit independent switching under different wavelengths, advancing multi‐photoswitch applications.

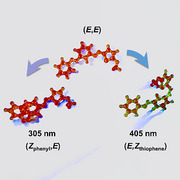

## 1 Introduction

**Figure 1:**
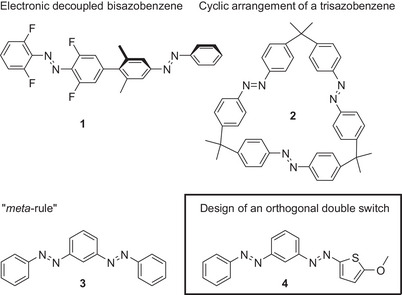
Bisazobenzenes **1**, separated by a linker.^[^ ^14^ ^]^ Trisazobenzene **2** arranged in a cyclic fashion.^[^ ^8^ ^]^ Independent switching by the “*meta*‐rule” **3**.^[^ ^16^ ^]^ Orthogonal switching in thiophenyl‐phenyl‐bis‐azobenzene **4**.

Manipulating molecules with high temporal and spatial resolution in a controlled way opens up tremendous opportunities in creating functional materials. Photoswitches exhibit this property, as they can change their structure upon irradiation, which can be applied with precise control in space and time. Hence, it is not surprising that molecular switches have been utilized in various areas, such as photopharmacology,^[^ ^1^ ^]^ catalysis,^[^ ^2^ ^]^ display technology,^[^ ^3^ ^]^ molecular wind‐up meter,^[^ ^4^ ^]^ information, and energy storage,^[^ ^5^ ^]^ or molecular machines.^[^ ^6^ ^]^ Nearly all photoswitches can adopt at least two distinct states allowing for reversible on‐off switching. The combination of multiple switches in one system can drastically increase the density of addressable functions and processable information. This can be realized either as a mixture of different single‐function switches^[^ ^7^ ^]^ or as integrated multi‐switches. The latter option further increases the density of functionality. To fully exploit the potential of such multi‐switch systems, it is essential to have the ability to selectively address individual units and access all possible states. Previously, we showed that positioning azobenzenes (ABs) in a cyclic arrangement alters the thermal stability of photoisomers and switching properties, which allows addressing different states selectively in a sequential fashion (**2**, Figure 1).^[^ ^8^ ^]^ Generally, orthogonal isomerization behavior can be achieved by incorporating different chemical switches into a single molecular entity. One example is the combination of photoswitches with acid/base switches.^[^ ^9^ ^]^ In such systems, however, the accessibility of certain states is often limited by the application of a correct sequence of stimuli, which leads to a *path‐dependent* isomerization behavior. The alternative of using light as the sole stimulus for orthogonal switching represents a major design challenge, requiring well‐separated absorption bands to selectively address each state with different colors of light. One option to realize such a system is the combination of two different photoswitches, as demonstrated previously using an AB together with a donor‐acceptor Stenhouse adduct (DASA) switch or an azobenzene‐oxindole dyad.^[^ ^10^ ^]^ The absorption bands of the different states can be further separated by altering the substitution pattern of the individual switches. For example, fluorine^[^ ^11^ ^]^ or methoxy^[^ ^12^ ^]^ substituents introduced in the *ortho*‐position of an AB enable (*E*)‐(*Z*)‐photoisomerization at 530 nm by shifting the *n*π* band of the (*E*)‐azobenzene bathochromically, thus allowing switching with visible light.^[^ ^13^ ^]^ Hecht and coworkers utilized this strategy and combined an alkyl‐substituted AB with a derivative bearing four fluorine atoms in the *ortho* position (**1**, Figure 1).^[^ ^14^ ^]^ A crucial aspect in their design was the introduction of methyl groups to maximize the torsion angle between the two AB switches to suppress any energy transfer between the chromophores, which is essential in the design of multi‐switch systems.^[^ ^15^ ^]^

Remarkably, we could show that independent switching properties can be maintained, even if two ABs share the same benzene core. In this case, a *meta*‐connectivity pattern is crucial to realize such a behavior (the so‐called “*meta*‐rule”, **3**, Figure 1).^[^ ^16^ ^]^ The strategy was also transferred to the combination of an azobenzene with a spiropyran.^[^ ^17^ ^]^ Here, also both switches share one benzene moiety. Depending on the connectivity pattern, different switching behaviors were observed. This concept enables a further increase in density of functionality within a double‐switch system, which has been used recently to design efficient bis‐azoarenes for molecular organic solar thermal systems (MOST).^[^ ^18^ ^]^ It has been shown computationally, that by adequate substitution of the AB arms, even a triple switch, in which each switching entity can be addressed individually, in principle is possible.^[^ ^19^ ^]^ Recently, we established, that thiophenyl‐azobenzenes (TphABs) exhibit considerable shifts of the absorption to the visible spectral region, enabling *E*‐*Z*‐isomerization at around ∼400 nm.^[^ ^20^, ^21^ ^]^ Combining a TphAB with an unsubstituted AB, which shows (*Z*)‐(*E*) isomerization in this spectral region, an alternating orthogonal switching behavior can be realized.

Combining this property with the “*meta*‐rule,” we designed a novel alternating thiophenyl‐phenyl‐bis‐azobenzene switch (MeO‐TphAB‐AB, **4**, Figure 1). In this work, we present the synthesis and the photophysics of this switch, supported by computations. This double switch is a bis‐azoarene, in which both AB arms share the central benzene ring, and yet, both AB moieties can be switched in an alternating orthogonal fashion. This is manifested by an alternating switching behavior due to the oppositional peaks of the monomers in their corresponding UV/Vis absorption spectra.

## 2 Results and Discussion

### 2.1 Synthesis

The targeted MeO‐TphAB‐AB **4** requires the connection of two different azo units to the same benzene core. Previous studies on azothiophenes have shown that the addition of zincated thiophenes to diazoniumbenzenes proceeds very efficiently, also in the presence of a large variety of functional groups.^[^ ^21^ ^]^ For the construction of unsymmetric ABs, the Bayer‐Mills reaction, as well as a Pd‐catalyzed coupling reaction of Boc‐protected hydrazines with aromatic halides, followed by oxidation, has been shown to work well.^[^ ^22^ ^]^

**Scheme 1:**
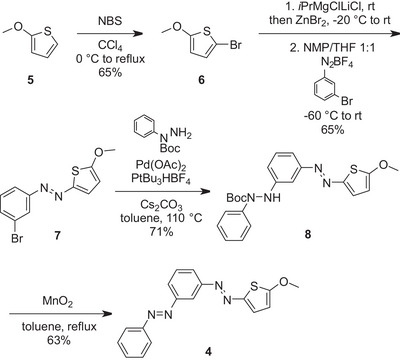
Synthesis of the target MeO‐TphAB‐AB **4** from commercially available starting materials in four linear steps.

Therefore, the synthesis was initiated by bromination of 1‐methoxythiophene (**5**) in 65% yield (Scheme 1). Halogen‐metal exchange with Knochel's reagent, followed by zincation, delivered the organometallic species required for the first key step: the addition of the metalated thiophene to the 3‐bromophenyldiazonium salt. For the installation of the second AB, the method reported by Cho and coworkers, successfully applied in the synthesis of star‐shaped ABs, was utilized, providing the target azoarene **8**.^[^ ^23^ ^]^ The usual protocol for the oxidation of *N*‐Boc‐diarylhydrazides using CuI and base at high temperature did not yield any of the desired product **4**. Therefore, 15 eq. of MnO_2_ and a short reaction time (15 minutes) had to be used to oxidize precursor **8** to obtain the targeted thiophenyl‐phenyl‐bis‐azobenzene compound **4**.

### 2.2 Switching Properties

**Figure 2:**
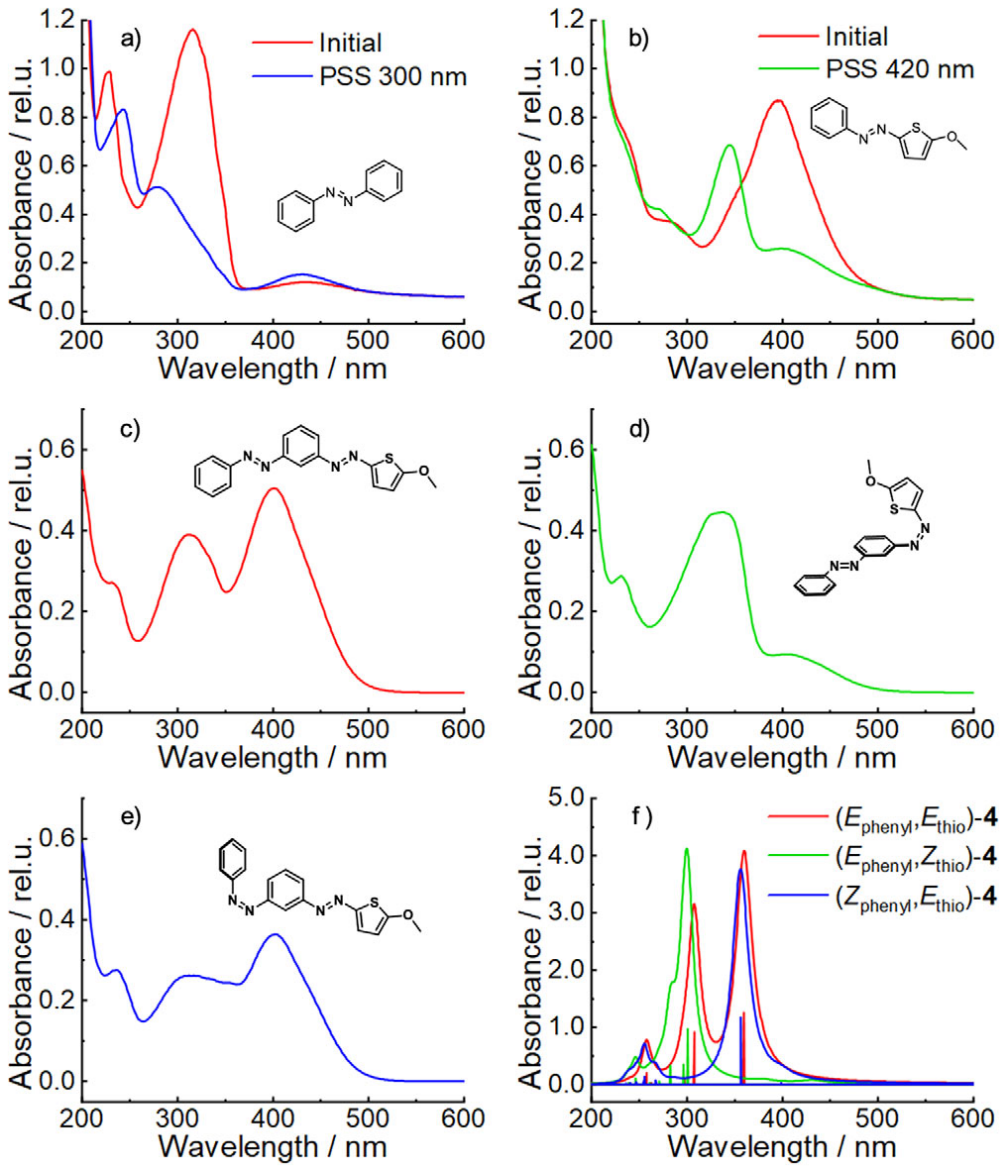
UV‐Vis irradiation experiments of monomers a) AB **9** and b) MeO‐TphAB **10** as well as MeO‐TphAB‐AB **4** in CH_3_CN, c) dissolved without illumination, d) photostationary state at 400 nm e) photostationary state at 300 nm. MeO‐TphAB‐AB **4** revealed orthogonal photoswitching. f) Computed electronic transitions support the observed behavior (for details see Supporting Information).

The thiophenyl‐phenyl‐bis‐azobenzene **4** exhibits two distinct absorption bands at around 400 nm as well as 320 nm (Figure 2c). These are found in the computed spectrum (Figure 2f) as well as in the monomers AB **9** (300 nm, Figure 2a) and MeO‐TphAB **10** (400 nm, Figure 2b) and are assigned to the ππ* absorption bands.^[^ ^21^ ^]^

The absorption bands of MeO‐TphAB‐AB **4** (Figure 2c) show no significant spectral shift compared to those of the monomers (Figure 2a,b), indicating that the coupling between the two photoswitchable units is small, in line with the *meta*‐connection (“*meta*”‐rule).^[^ ^16^, ^24^ ^]^ Therefore, orthogonal switching of the two units is expected. Irradiation with 400 nm depletes the band at 400 nm (Figure 2d), which can be rationalized by the selective (*E*)→(*Z*) isomerization of the TphAB unit. On the contrary, upon irradiation at 305 nm, the absorption at 320 nm is reduced due to the (*E*)→(*Z*) isomerization of the AB moiety. However, a small reduction of the MeO‐TphAB ππ* band is also detected, denoting isomerization of some MeO‐TphAB units.

^1^H‐NMR spectroscopy was employed to quantify the degree of switching, as well as the thermal relaxation behavior. Two series of NMR data are shown in Figures S4 and S5: One dataset was acquired with sample irradiation with 415 nm during NMR measurement (in situ)^[^ ^25^ ^]^ followed by thermal relaxation (Figure S4), and one dataset with alternating irradiation at 415 nm inside the probe (in situ) and at 310 nm outside the magnet (ex situ) (Figure S5). As expected, the initial state showed almost exclusively the (*E*_phenyl_ *,E*_thio_) isomer (≥97%, see Figure S4). Upon irradiation with 415 nm (in situ), a photo‐stationary state (PSS) was reached, in which the (*E*_phenyl_ *,Z*_thio_) isomer is the main component with 67% relative concentration (see Table S3). In contrast, irradiation with 310 nm leads to a PSS with a large population of the (*Z*_phenyl_ *,E*_thio_) isomer (28% *Z*_phenyl_ *,E*_thio_; 55% *E*_phenyl_ *,E*_thio_), thereby realizing an alternating orthogonal switching process.

The relatively low content of the (*Z*_phenyl_ *,E*_thio_) isomer in the PSS generated by 310 nm irradiation indicates, that the AB cannot be addressed selectively at the chosen wavelength. To understand this finding, possible energy transfer mechanisms need to be investigated. Therefore, a combination of transient absorption measurements and electronic structure calculations was conducted.

### 2.3 Transient Absorption Measurements

**Figure 3:**
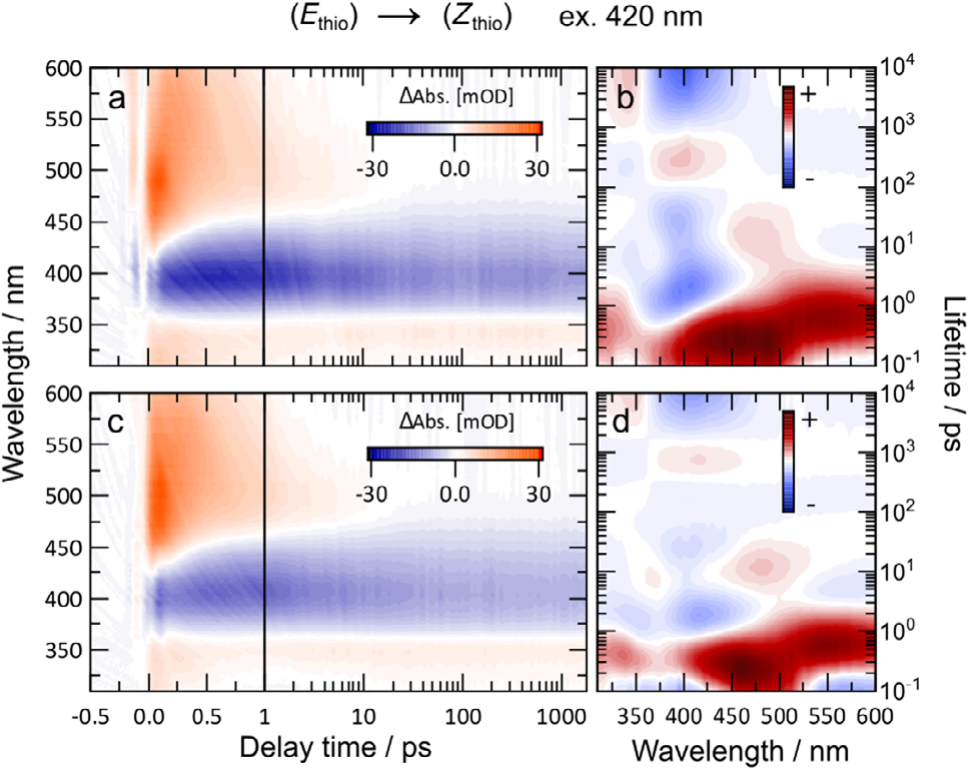
(*E*_thio_)→(*Z*_thio_) isomerization of MeO‐TphAB **10** (a, b) and MeO‐TphAB‐AB **4** (c, d), triggered by excitation pulses of 420 nm. The time‐resolved transient maps are presented in a, c and the lifetime density maps are presented in b, d.

**Figure 4:**
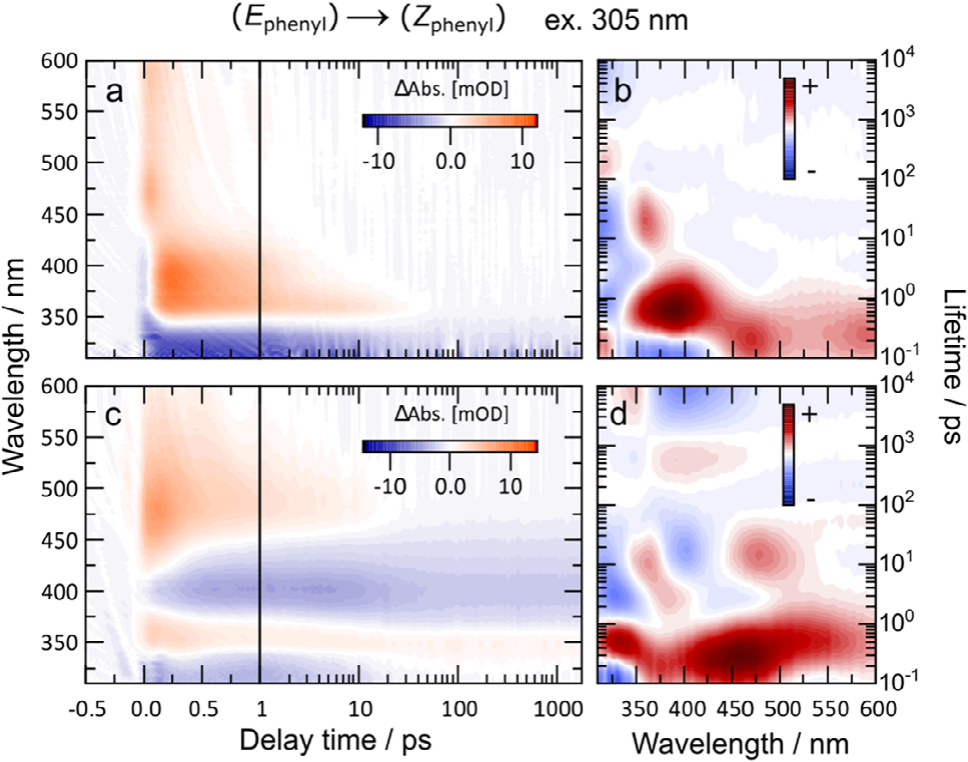
(*E*_phenyl_)→(*Z*_phenyl_) isomerization of azobenzene (a, b) and MeO‐TphAB‐AB **4** (c, d), triggered by 305 nm excitation pulses. The time‐resolved transient maps are presented in a and c. The lifetime density maps are presented in b and d.

The orthogonal (*E*)→(*Z*) switching of the two AB arms was investigated by selectively exciting the ππ* bands of the AB subunit and nπ* band of the MeO‐TphAB subunit in **4**. The resulting time‐resolved transient absorption spectra were compared to their corresponding monomers (Figure 3 for MeO‐TphAB and Figure 4 for AB). Furthermore, the (*E*_phenyl_,*Z*_thio_)→ (*E*_phenyl_ *,E*_thio_) isomerization of MeO‐TphAB was investigated with 340 nm and 305 nm excitation.

Upon 420 nm excitation, the photoinduced dynamics of **4** are nearly identical to that of the MeO‐TphAB **10** monomer (Figure 3a,c) and the lifetime analyses are also strongly correlated, indicating the individual excitation and switching of the MeO‐TphAB unit in **4**. Their excited state dynamics (*E*_thio_)→(*Z*_thio_) resemble what was previously reported in thiophenylazobenzene (TphAB) without the methoxy group.^[^ ^20^ ^]^ The only noteworthy difference is that the ground state bleach (GSB) signal from 350 nm to 450 nm of MeO‐TphAB **4** has a bathochromic shift compared to unsubstituted TphAB, which leads to partial compensation with the excited state absorption (ESA) signal in the 400–450 nm range before 10 ps. The pronounced ESA band (450–500 nm), corresponding to the initially excited ππ* state, decays with about 200 fs to populate the nπ* excited state (ESA above 500 nm). These two processes are described by two distinct lifetimes in the same wavelength range at 0.2 ps and 0.8 ps, respectively (Figure 3b,d).

Below 350 nm, another positive signal could be assigned to the excited‐state absorption from the nπ* state, which later results in the formation of the (*Z*)‐isomer product absorption band. Notably, the nπ* excited state relaxes with a time constant of 0.8 ps and leads to the formation of hot ground‐state (HGS) (*E*)‐isomers, visible as a blue‐shifted elongated positive signal toward the GSB slightly below 500 nm. The lifetime analysis shows that the HGS is mediated by the dynamics around the conical intersection, which could be described by the negative amplitude around 420 nm, centered around 1–2 ps (Figure 3b,d). The cooling dynamics of the HGS molecules can be described by a 10–20 ps lifetime, which partially repopulates the (*E*)‐isomers. Note that the dominant 400 nm ππ* band of the MeO‐TphAB unit in **4**, which is more than 10‐fold more intense than the nπ* transition band of the AB unit, conceals any potential contribution from excitation of the AB unit in **4**.

Excitation at 305 nm addresses predominantly the ππ* band of the AB unit of **4**. For comparison, we show the well‐studied dynamics of the AB monomer **9** in Figure 4a.^[^ ^26^ ^]^ After ππ* excitation of (*E*)‐AB **9** we observe the ππ* ESA band at 450–500 nm, which decays with about 100 fs lifetime, which in turn decays to form the nπ* ESA at 350–430 nm. The AB (*E*)→(*Z*) isomerization occurs from this state with an overall lifetime of ∼700 fs (broad lifetime distribution) and leads to the formation of HGS *E‐*isomers (350–375 nm), which cool off with 10–20 ps lifetime. Unexpectedly, we observe that the dynamics of the MeO‐TphAB‐AB **4** after excitation with light of 305 nm shows contributions of both the AB and MeO‐TphAB dynamics (Figure 4c). This is clearly visible in the experimental data as well as the lifetime distribution maps (Figure 4d). Given the relatively low absorption contribution of the MeO‐TphAB (in MeO‐TphAB‐AB **4**) at 305 nm, we conclude that some ultrafast energy redistribution occurs toward MeO‐TphAB resulting in its isomerization. This interpretation is consistent with the lower than expected levels of (*Z*_phenyl_ *,E*_thio_)‐isomer observed in the ^1^H‐NMR spectra following 310 nm irradiation.

Aside from addressing the selective (*E*)→(*Z*) isomerization of the two units in **4**, the photoinduced (*Z*_thio_)→(*E*_thio_) isomerization of the MeO‐TphAB units was studied to explore the capability of photo‐control. The intuitive choice is to excite the product absorption band (Figure 2d) of (*Z*)‐MeO‐TphAB **4** at 340 nm, as the product band has a relatively high extinction coefficient and the steady‐state measurements showed significant amounts of (*E*)‐isomers in the photostationary state at 340 nm.

**Figure 5:**
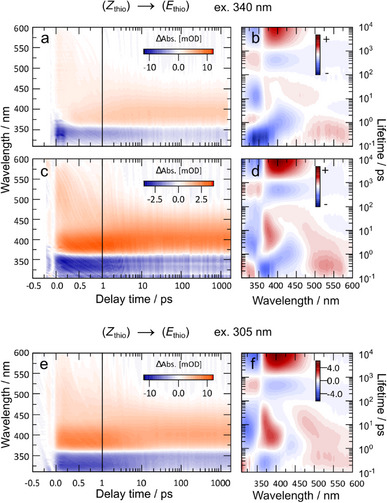
(*Z*_thio_)→(*E*_thio_) isomerization of the MeO‐TphAB monomer **10** (a, b) and bis‐AB‐TphAB **4** (c, d, e, f), triggered by different excitation wavelengths. The left column presents the transient absorption data, and the right column presents the corresponding lifetime density maps.

Following this strategy, the (*Z*_thio_)→(*E*_thio_) reaction dynamics were investigated by exciting both samples (**10** and **4**) with 340 nm pulses, while the samples were constantly irradiated with a 400 nm LED lamp to populate the *Z‐*isomers of the MeO‐TphAB subunits. The transient absorption map of monomer **10** presents a broad, weak ESA at the picosecond time scale from 400 nm to 600 nm, which was assigned to the relaxation on the nπ* surface (Figure 5a). This ESA later narrows into a blue‐shifted vibrationally HGS signal after around 10‐20 ps at 450‐500 nm, which later forms the product absorption of the *E*‐isomer at 400 nm. Note that this HGS resembles the HGS signal in the reverse (*E*_thio_)→(*Z*_thio_) isomerization of **10**, indicating a similar cooling process after passage of the conical intersection. The (*Z*_thio_)→(*E*_thio_) isomerization generally presents an inverted signal of (*E*_thio_)→(*Z*_thio_) after passing the conical intersection, as the HGS develops into the product band rather than GSB recovery. The GSB recovery for (*Z*_thio_)→(*E*_thio_) could be observed at the same timescale as the (*Z*)‐isomers are populated by a weak HGS at 375 nm. It is worth mentioning that the higher excited state dynamics are not observed even with higher excitation energy, compared to the measurements of (*E*_thio_)→(*Z*_thio_) isomerization (Figure 4a,c), due to the ultrashort relaxation overlapping with the GSB. Furthermore, the S_1_ relaxation is slightly shorter compared to the (*E*_thio_)→(*Z*_thio_) isomerization process. The double switch **4** presents nearly identical dynamics compared to the measurements of monomer **10**, with the exception that there is a strong ESA band appearing between 375 nm and 425 nm until 10 ps, which is assigned to the nπ* absorption and the HGS of the AB units in **4** (Figure 5c). This occurrence of the AB excited state signal is due to the overlap of the absorption band of (*Z*)‐isomer of MeO‐TphAB and the ππ* absorption band of AB. This ESA is visible with strong positive elongated lifetime amplitudes from 350 nm to 400 nm centered around 10 ps (Figure 5d). Compared to the same lifetime amplitude of the nπ* ESA from AB (Figure 5b), the lifetime components from **4** present a narrow and nonexponential shape, due to the superimposition of the nπ* excited state of AB and the HGS signals of MeO‐TphAB and AB. The prolonged negative lifetime distribution of the GSB recovery below 350 nm from 1 to 100 ps reflects these multiexponential processes (Figure 5d). To further support this observation, we excited **4** with 305 nm pulse to S_4_ under the same experimental condition (Figure 5e,f). The 305 nm excitation should yield more (*E*_phenyl_)→(*Z*_phenyl_) conversion of AB units rather than (*Z*_thio_)→(*E*_thio_) isomerization of MeO‐TphAb units according to the steady‐state measurements. The results expectedly show a more intensive ESA between 370 nm and 420 nm (Figure 5e), and a more intensive amplitude of the lifetime of the nπ* ESA from AB around 3 ps (Figure 5f). It is worth to mention that, the 700 fs ESA for ππ* of AB is not observed in any transient experiments of **4**, which again indicates energy redistribution upon excitation to the states higher than nπ* state of both subunits.

### 2.4 Computations

**Figure 6:**
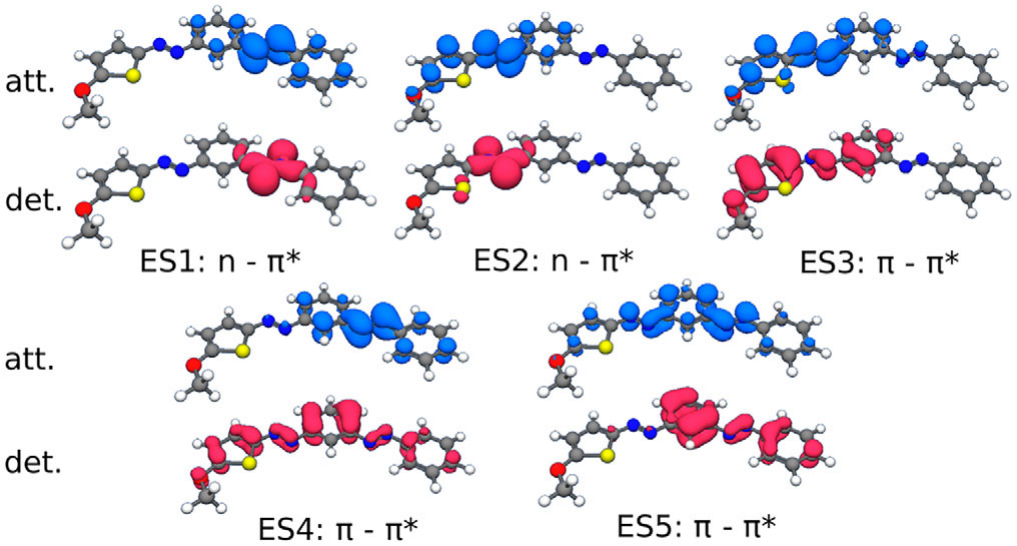
Detachment (red) and attachment (blue) densities of the first five excited states of **4** at the CAM‐B3LYP‐D3(BJ)/6–311G* level of theory.

The vertical excited states have been computed at TDDFT/CAM‐B3LYP‐D3(BJ)/6–311G* level. It has been shown previously that this level of approximation is sufficient to investigate the photochemistry of multiazobenzene derivatives with qualitative sometimes even quantitative agreement with experiment and higher levels of theory.^[^ ^17^, ^20^ ^]^ The computed S_1_ and S_2_ states of (*E*_phenyl_ *,E*_thio_)‐**4** exhibit excitation energies of 2.81 and 3.03 eV, respectively. Their corresponding detachment and attachment density plots (Figure 6) identify them as the typical nπ* states and show the S_1_ to be located exclusively on the AB branch of (*E*_phenyl_ *,E*_thio_)‐**4**, while the S_2_ is confined to the MeO‐TphAB branch. Since the photo‐induced isomerization of AB derivatives proceeds largely in the nπ* state, this finding supports the electronic orthogonality of the nπ* states of the two branches. The S_3_ of (*E*_phenyl_ *,E*_thio_)‐**4** has a calculated excitation energy of 3.66 eV, corresponds to the typical ππ* state and is strictly localized on the TphAB branch. The situation is different for the closely spaced S_4_ and S_5_ states, at 4.19 and 4.26 eV, respectively. While these states have ππ* character, they are not strictly localized at the AB branch of (*E*_phenyl_ *,E*_thio_)‐**4**. Their detachment and attachment densities extend also to the MeO‐TphAB branch (Figure 6). Since S_3_, S_4,_ and S_5_ correspond to the initially excited states in the switching experiments, individual switching of the MeO‐TphAB unit may be possible in **4** upon excitation of S_3_, while excitation of S_4_ and S_5_ may lead to predominant switching of the AB branch but also the MeO‐TphAB unit, which is seen in the transient absorption experiments.

**Figure 7:**
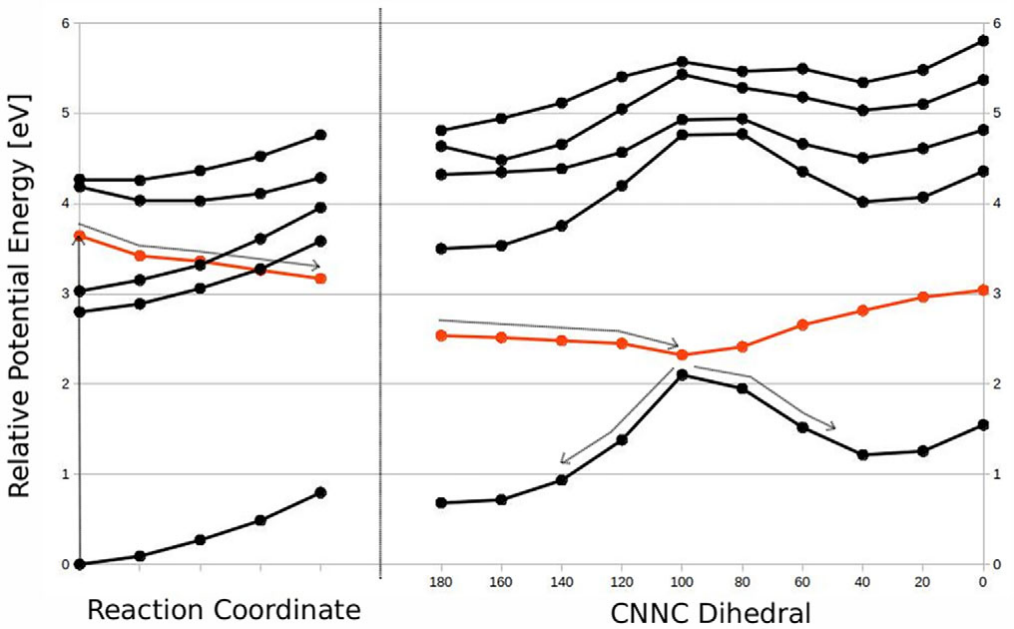
Isomerization pathway of S_3_. Relaxed scan of the potential energy surface along the isomerization coordinate of the TphAB branch of **4**. Left: Potential energy surfaces along the unconstrained optimization of S_3_, the ππ* state on the TphAB branch, decaying nonradiatively to become the S_1_. Right: Potential energy surface scan of the relaxed nπ* state of the TphAB branch along the CNNC dihedral angle. The red lines correspond to the optimized state. Dotted arrows indicate the excitation and the path followed on the PES.

To further corroborate whether excitation into the optically bright ππ* states leads to individual photoswitching of the branches, relaxed scans along the excited state (*E*_phenyl_,*E*_thio_)→(*E*_phenyl_,*Z*_thio_) isomerization coordinate, that is, approximated as the CNNC dihedral angle of the corresponding branch, were performed (Figure 7). Starting in the S_3_ state, the MeO‐TphAB unit relaxes quickly to reach the S_1_ state already at the initial dihedral angle of 180° and undergoes isomerization from (*E*_phenyl_,*E*_thio_)→(*E*_phenyl_,*Z*_thio_) without an energy barrier by rotation of the CNNC dihedral located at the ThAB.

In contrast, starting the relaxation from the S_4_ state does not lead to such an immediate conversion to S_1_ (Figure 7). Instead, the initial geometry optimization yields a flat excited‐state minimum. This behavior extends the lifetime of S_4_ compared to S_3_. In effect, due to the delocalization of the S_4_ over both branches of **4**, the AB and the MeO‐TphAB branches are populated to a certain extent, leading to isomerization of both branches.

## 3 Conclusion

In conclusion, the bis‐azobenzene‐thiophenylazobenzene MeO‐TphAB‐AB‐**4** was designed to show orthogonal switching behavior, as both the thiophenyl‐AB and the phenyl‐AB moieties exhibit different ππ* transition energies. A successful synthesis strategy of MeO‐TphAB‐AB‐**4** was subsequently implemented, and UV‐Vis spectroscopy revealed two distinct ππ* bands separated from each other at 320 and 400 nm. By irradiation into these absorption maxima, an enrichment of the respective isomer (*Z*_phenyl_ *,E*_thio_) or (*E*_phenyl_ *,Z*_thio_) was achieved, leading to alternating switched states. Ultrafast time‐resolved experiments confirmed the individual switching of the MeO‐TphAB unit in **4**, however, mixed dynamics were seen when exciting **4** with higher photon energy. Lifetime analysis reveals a combination of the nπ* dynamics of AB and MeO‐TphAB, indicating independent isomerization processes through the conical intersection. Furthermore, TDDFT calculations showed that S_1_ and S_2_ are nπ* states, located exclusively on the AB (S_1_) and MeO‐TphAB branches (S_2_), while S_3_, S_4,_ and S_5_ were ππ* excitations. While S_3_ is exclusively localized on the MeO‐TphAB moiety, S_4_ and S_5_ are partially delocalized over both moieties. Relaxed scans along the excited‐state isomerization pathway of the MeO‐TphAB unit support its individual switching ability, when excited into the S_3_ state. The AB subunit, on the other hand, is not entirely individually switchable due to the delocalization of the S_4_ and S_5_ states also onto the MeO‐TphAB unit. This deep understanding of the photochemical principles for individual photoswitching paves the road for future design of orthogonal photoswitches toward their usage as information storage systems and further applications.

## Supporting Information

Additional references cited within the Supporting Information.^[^ ^27^, ^28^, ^29^, ^30^, ^31^, ^32^, ^33^, ^34^ ^]^

## Conflict of Interest

The authors declare no conflict of interest.

## Supporting information



Supporting Information and https://doi.org/10.5281/zenodo.16222845.

## Data Availability

The data that support the findings of this study are available in the supplementary material of this article. The data used for characterizing the photoswitching by NMR have been deposited at https://doi.org/10.5281/zenodo.16222845.
