# Supplementary material for: Alternating Orthogonal Switching in a Thiophenyl‐Phenyl‐Bis‐Azobenzene Switch
Source: Chemistry. 2025 Aug 22;31(57):e01976. doi: 10.1002/chem.202501976 (PMC12520053; doi:10.1002/chem.202501976)
Supplement: Supplementary file 1 — Supporting Information and https://doi.org/10.5281/zenodo.16222845. [file CHEM-31-e01976-s001.pdf]

Supporting Information

General Information 2
  Synthesis and Characterization.. 2
  Irradiation Experiments 3
Synthesis 6
^{1}H and ^{13}C{^{1}H} NMR Spectra 10
UV-Vis Spectroscopy 13
^{1}H-NMR Isomerization experiments.. 15
Computational Methods 23
Ultrafast Time-resolved Spectroscopy 29
  Ultrafast Time-resolved Measurements in DMSO 29
References………………………………………………………………………33

General Information

Synthesis and Characterization

Chemicals were used as purchased from Sigma-Aldrich, Acros Organics, Alfa Aesar and TCI Europe. Anhydrous solvents were purchased from Acros Organics. Technical grade solvents used during workup and purification were distilled prior to use. Air and/or water-sensitive reactions were carried out under Schlenk-conditions. Solids were dried under high vacuum (oil pump, ca 10^{-3} mbar) at rt, 50 °C or 60 °C when necessary. Flash column chromatography and column chromatography was carried out with Silica 60 M (0.04 – 0.063 mm) or Silica 60 (0.063 – 0.2 mm) from Macherey Nagel GmbH & Co. KG. Medium-pressure liquid chromatography (MPLC) was carried out on a Büchi Sepacore® flash chromatography system X-50 with Sepacore® Flash cartridges (particle size: 40–63 µm). Thin layer chromatography was performed on Polygram® SIL G/UV_{254} from Macherey Nagel GmbH & Co. KG. NMR spectra were measured on a Bruker Avance II 200 MHz, Avance II 400 MHz, Avance III 400 MHz HD or Avance III 600 MHz spectrometer at room temperature, if not stated otherwise. Chemical shifts are reported in parts per million (ppm) relative to the solvent peak, coupling constants (*J*) are reported in Hertz (Hz). Deuterated solvents were obtained from Deutero GmbH (Kastellaun, Germany) or Euriso-Top GmbH. For all azobenzenes, the thermodynamically more stable (*E*)-isomer is reported if not noted otherwise. ESI-MS spectra were recorded on a Bruker Daltonics Micro TOF. APCI mass spectra were obtained by using the same system with a Bruker APCI II ion source. Melting points were measured on a Krüss M5000 capillary melting point meter with a heating rate of 1°C/min.

Irradiation Experiments

Irradiation of UV/Vis samples was conducted in an in-house built box (Figure S1) using LEDs by Lumitronix or Nichia (see Table S1). After the given irradiation times, the samples were immediately placed and measured in the corresponding spectrometer if not noted otherwise.

For NMR experiments with sample irradiation, we used a NMR sample chamber equipped with a glass fiber for in-situ illumination, akin to the setup described by Feldmeier *et al*.[27] For irradiation with 415 nm, light was coupled into the NMR sample tube using a glass fiber, and the sample was irradiated, while the sample was located inside the NMR probe (in-situ illumination). The LED was centered onto the flat-cut end of a multimode glass fiber (FT100UMT, Thorlabs, Inc.). The glass fiber was roughened at the opposing end with sand paper, and inserted into a 3.3 mm outer diameter (OD) coaxial insert (WGS-5BL-SP, Wilmad Labglass, Inc.). Samples of 280 µL, containing 5 mM of **4** in DMSO-d_6, were transferred into a 4.2 mm inner diameter (ID) NMR tube (5.0 mm OD, ATS Life Sciences Wilmad, 507-PP-7) and the insert containing the glass fiber was immersed into the solution for measurements. ^{1}H-NMR spectra with in-situ illumination were collected during continuous illumination.

Irradiation at 310 nm was performed outside of the NMR spectrometer (ex-situ), with intermittent transfer of the sample into the spectrometer, for spectrum measurement without illumination during the measurement. The sample cell used for in-situ illumination with 415 nm remained assembled during the ex-situ illumination, and the sample region was illuminated from the outside with one 310 nm LED, placed at a distance of 2 cm from the LED. For ex-situ illumination, sample transfer between the illumination device and the spectrometer was performed manually. Cycles of manual transfer to the illumination device (roughly 15 s), illumination with 310 nm for 30 s, back-transfer to the NMR spectrometer (roughly 15 s) and 30 s of sample equilibration and spectrum acquisition were repeated to monitor the photoisomerization process.

For constructing the time-axis shown in Figure S5, in the case of ex-situ illumination only the periods of actual sample illumination were used to increment the time axis. Periods of sample transfer or intermittent ^{1}H-NMR measurements were not added. Similarly, the time period for replacing a broken NMR tube (note “b)” in Figure S5; duration: 20 min) was not added to the time-axis. All other time periods (without

illumination or with in-situ illumination), were directly added to time-axis without modification.

A Lumiled LUXEON LHUV-0415-A070 SMD LED, driven at 350 mA constant current was used as light source for irradiation at 415 nm. For irradiation at 310 nm an ILR ILR-XN01-S300-LEDIL-SC201 SMD LED was used. At 350 mA constant current, the performance of the 310 nm LED, as measured using an integration sphere (Avantes AvaSphere-50) coupled to a spectrophotometer (Avantes AvaSpec-3648), was found to be unstable. Therefore, a pulse width modulator (LUMITRONIX 50975.01) was used to dim the LED, until the point, where the output intensity remained stable over the course of 1 h. In all cases, 350 mA constant power sources from Eaglerise Co., Ltd. (ELP10X1PS) were used.

NMR data with sample illumination was collected on an Avance III HD spectrometer (Bruker Biospin GmbH) operating at 400.13 MHz ^{1}H frequency, equipped with a 5 mm broadband observe (BBO) probe (BB{^{19}F – ^{109}Ag}-^{1}H/^{2}H) with a z-gradient. Sample temperature was controlled at 300 K during NMR measurements, whereas no active temperature control was applied during ex-situ irradiation. ^{1}H-NMR spectra series with 30° flip-angle pulses (3.67 µs), followed by 0.8192 s acquisition and a 0.25 s recycling delay ($TR = 1.0692\ s$) were acquired. For the spectra shown in panels B & D in Figure S4), spectra with 8 dummy scans and 128 scans were acquired (145.4 s per spectrum). For all other data shown in Figures S4 & S5, continuous sampling was used without dummy scans, and eight scans per spectrum were collected resulting in 8.6 s per spectrum.

To compensate for systematic errors of the molar fractions of the stereoisomers caused by fast pulsing, measured integrals were normalized by the steady-state signal saturation factor f,[28] obtained under the assumption of fully isolated sets of isochronous spins and fast transverse decoherence ($TR \gg T_2^*$)

$$f(TR, T_1, \beta) \approx \frac{1-\exp\left(-\frac{TR}{T_1}\right)}{1-\cos(\beta)\exp\left(-\frac{TR}{T_1}\right)}.$$

Here TR ($1.0692\ s$) is the time between two consecutive pulses of flip-angle β ($30°$) and T_1 is the relaxation time constant of the respective signal. T_1 relaxation time constants of the methoxy-groups used for quantification were measured for all four stereoisomers, using an inversion-recovery experiment with continuous irradiation at 415 nm (see Table S2).

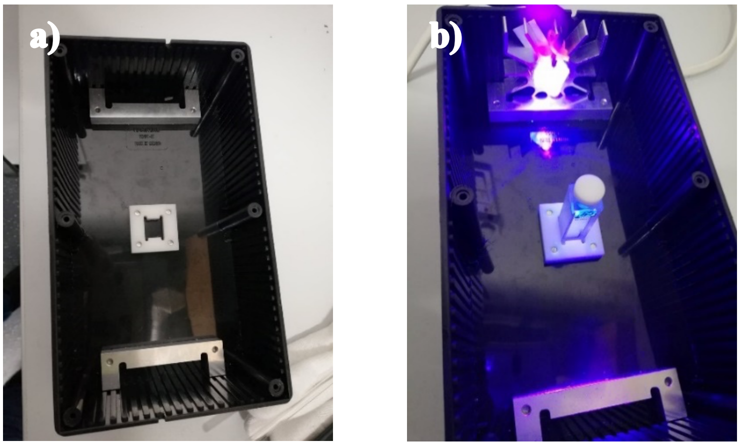

Figure S1: In-house built LED irradiation box for UV/Vis cuvettes. a) Box without LED, cuvettes, b) irradiation of cuvettes.

Table S1: LEDs used for all irradiation experiments with their specifications.

λ_{max} / nm	$\Delta\lambda_{FWHM}$ / nm	Product name
256	n.a.	3UV-38 handheld lamp (8 W)
302	n.a.	3UV-38 handheld lamp (8 W)
310	13	ILR-XN01-S300-LEDIL-SC201
365	9	NCSU276AT-U365
385	10	NCSU276AT-U385
405	12	NCSU276AT-U405
415	16	LHUV-0415-A070
425-430	14	LHUV-0425-0650
448	20	LXML-PR01-0500
470	20	LXML-PB01-0030

Table S2: T_1-values measured for the methoxy ^{1}H-NMR signals of **4** in DMSO-d_6, and steady-state signal saturation factor f obtained at the experimental parameters used.

isomer	T_1(^{1}H, **4**, -OMe) [s]	$f(TR = 1.0692\ s, T_1, \beta = 30°)$
(E_{phenyl},E_{thio})	1.21 ± 0.02	0.914
(Z_{phenyl},E_{thio})	1.41 ± 0.05	0.894
(Z_{phenyl},Z_{thio})	1.37 ± 0.03	0.898
(E_{phenyl},Z_{thio})	1.33 ± 0.02	0.902

Synthesis

Synthesis of Precursors

5-Bromo-2-methoxythiophene **6**, 3-bromobenzene-diazonium tetrafluoroborate and phenyl-*N*-Boc-hydrazide was synthesized according to the literature.[29]

***Meta*-bromophenyl-5-methoxyazothiophene 7**

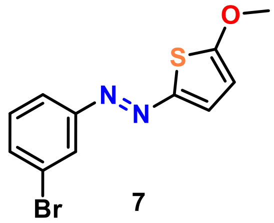

7

In a dry Schlenk-tube under a nitrogen atmosphere, *i*-PrMgCl · LiCl (1.3 M, 2.6 mL, 3.4 mmol, 2.0 equiv.) in THF was added dropwise to a solution of 2-bromo-5-methoxythiophene **6** (0.31 mL, 2.6 mmol, 1.5 equiv.) in dry THF (1.7 mL) at rt. The solution was stirred at rt for 2.5 h and was cooled to –20 °C afterwards. Following, $ZnBr_2$ in dry THF (1.0 M, 1.4 mL, 1.4 mmol, 0.82 equiv.) was added dropwise and the solution was allowed to warm rt and was stirred at rt for 45 min. Then, the dithiophenylzinc solution was added dropwise to a suspension of 3-bromobenzene-diazonium tetrafluoroborate (510 mg, ca 90%, 1.69 mmol, 1.00 equiv.) in a mixture of dry THF and dry NMP (3.7 mL and 1.7 mL) at –60 °C under a nitrogen atmosphere. More THF (5 mL) was added to facilitate stirring, which, however, had no effect. After complete addition, the suspension was slowly warmed to rt and stirred for 2 h. After dilution with CH_2Cl_2 (20 mL), the reaction was quenched by the consecutive addition of sat. aq. NH_4Cl (10 mL) and water (10 mL). After phase separation, the aqueous phase was extracted with CH_2Cl_2 (3 x 10 mL) and the combined organic phases were dried over $MgSO_4$, filtered and the solvents were evaporated under reduced pressure to yield a red solid, which was purified by flash column chromatography (SiO_2, cyclohex/EtOAc; 10:1) to yield a dark red solid (328 mg, 65%).

**^{1}H-NMR**	(600 MHz, $CDCl_3$) δ 7.92 (t, *J* = 1.9 Hz, 1H), 7.73 – 7.68 (m, 1H), 7.56 (d, *J* = 4.4 Hz, 1H), 7.50 – 7.45 (m, 1H), 7.32 (t, *J* = 7.9 Hz, 1H), 6.35 (d, *J* = 4.4 Hz, 1H), 3.99 (s, 3H).
**^{13}C-NMR**	(151 MHz, $CDCl_3$) δ 172.1, 153.4, 146.8, 133.8, 132.3, 130.5, 124.4, 123.2, 122.3, 105.8, 60.1.

HRMS (ESI) m/z for $[M+H]^+$; calcd. 296.9692, found 296.9710.

Melting Point (°C) 82

Meta-thiophenylazo-phenyl-_N_-Boc-hydrazide 8

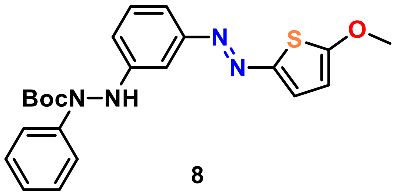

8

In an oven-dried Schlenk-tube under nitrogen atmosphere, 3-bromophenyl-azo-5-methoxythiophene **7** (141 mg, 474 μmol, 1.00 equiv.), *N*-Boc-phenylhydrazide (110 mg, 528 mg, 1.11 equiv.), $Pd(OAc)_2$ (17 mg, 74 μmol, 15 mol%), *t*-BuP · HBF_4 (23 mg, 78 μmol, 16 mol%) and Cs_2CO_3 (237 mg, 720 μmol, 1.52 equiv.) were suspended in dry toluene (1 mL). After stirring the suspension for 30 min at rt, the tube was sealed and the reaction mixture was stirred at 110 °C for 5 h. After cooling to rt, the mixture was filtered through a silica plug (washed with EtOAc) and was purified by column chromatography (SiO_2, cyclohex/EtOAc; 5:1) to yield a red oil (150 mg, contains 5% inseparable *N*-Boc-phenylhydrazide, 71%).

**^{1}H-NMR**	(400 MHz, $CDCl_3$) δ 7.65 – 7.58 (m, 2H), 7.50 (d, *J* = 4.4 Hz, 1H), 7.38 – 7.28 (m, 4H), 7.27 – 7.24 (m, 1H), 7.15 – 7.10 (m, 1H), 6.86 – 6.79 (m, 1H), 6.46 (s, 1H), 6.31 (d, *J* = 4.4 Hz, 1H), 3.96 (s, 3H), 1.37 (s, 9H).
**^{13}C-NMR**	(101 MHz, $CDCl_3$) δ 171.2, 154.0, 153.3, 149.3, 147.1, 142.9, 132.6, 129.8, 128.7, 124.7, 121.8, 116.1, 114.5, 106.4, 105.3, 82.6, 60.0, 28.2.
**HRMS (ESI)**	m/z for $[M+H]^+$; calcd. 425.1642, found 425.1641.

1-Phenyl-3-(5-methoxythiophenyl)bisazobenzene 4

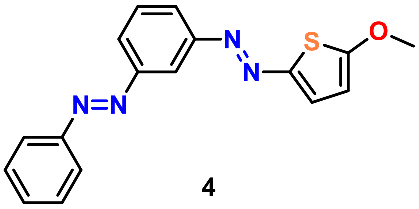

4

To a solution of azo-phenylhydrazide **8** (126 mg, 95%, 282 μmol, 1.00 eq.) in dry toluene (5 mL), activated MnO_2 (88%, 418 mg, 4.23 mmol, 15.0 eq.) was added and the suspension was stirred at reflux for 35 min. After cooling to rt, the mixture was filtered through a silica plug (washed with CH_2Cl_2) and was concentrated under reduced pressure. The crude product **4** was purified by flash column chromatography (SiO_2, cyclohex/EtOAc/DCM; 20:1:1) to yield an orange solid (57 mg, 63%).

**^{1}H-NMR**	(400 MHz, $CDCl_3$) δ 8.31 (t, *J* = 1.9 Hz, 1H), 7.98 – 7.93 (m, 3H), 7.90 (d, *J* = 1.1 Hz, 1H), 7.64 – 7.57 (m, 2H), 7.57 – 7.46 (m, 3H), 6.36 (d, *J* = 4.4 Hz, 1H), 4.00 (s, 3H).
**^{13}C-NMR**	(101 MHz, $CDCl_3$) δ 171.8, 153.6, 153.2, 152.8, 147.1, 133.4, 131.4, 129.7, 129.3, 125.1, 124.4, 123.1, 115.9, 105.6, 60.1.
**HRMS (ESI)**	$[M+Na]^+$; calcd. 345.0780, found 345.0782.

^{1}H- and ^{13}C{^{1}H}-NMR Spectra

***Meta*-bromophenyl-5-methoxyazothiophene 7**

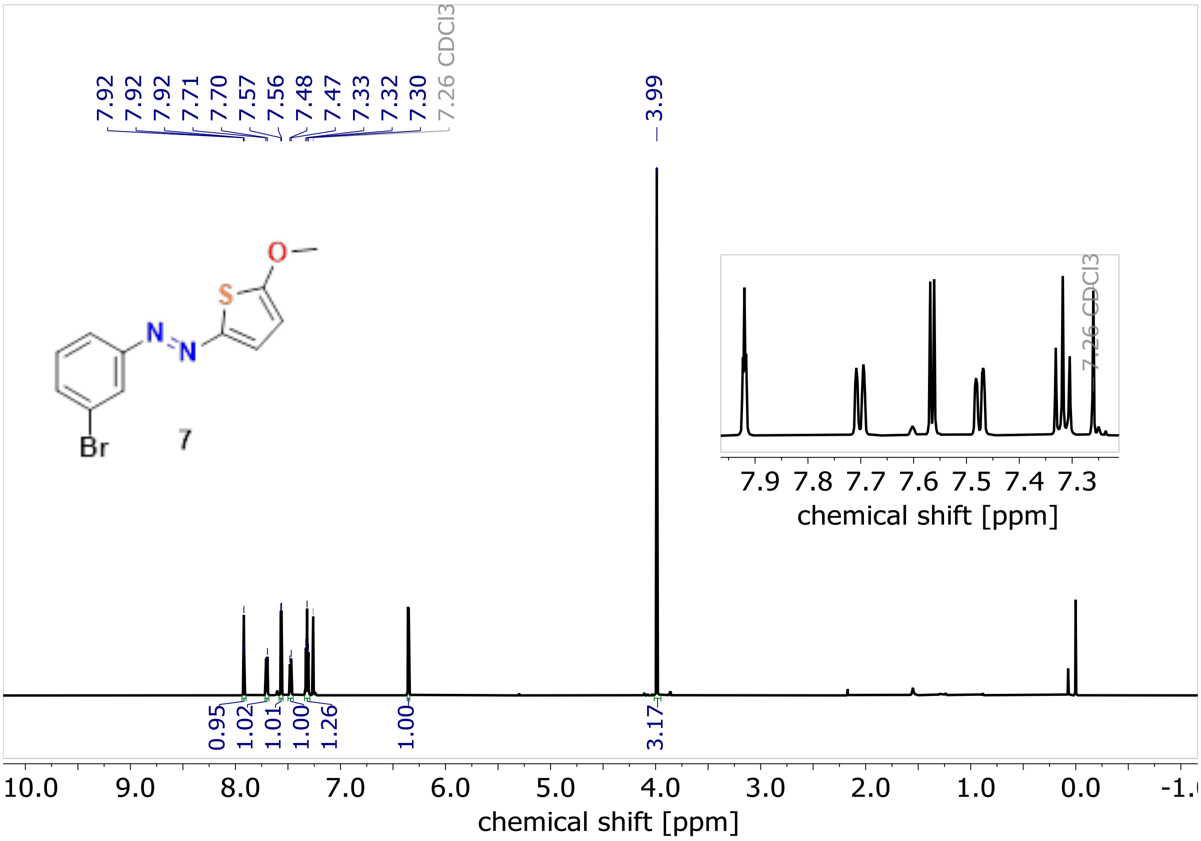

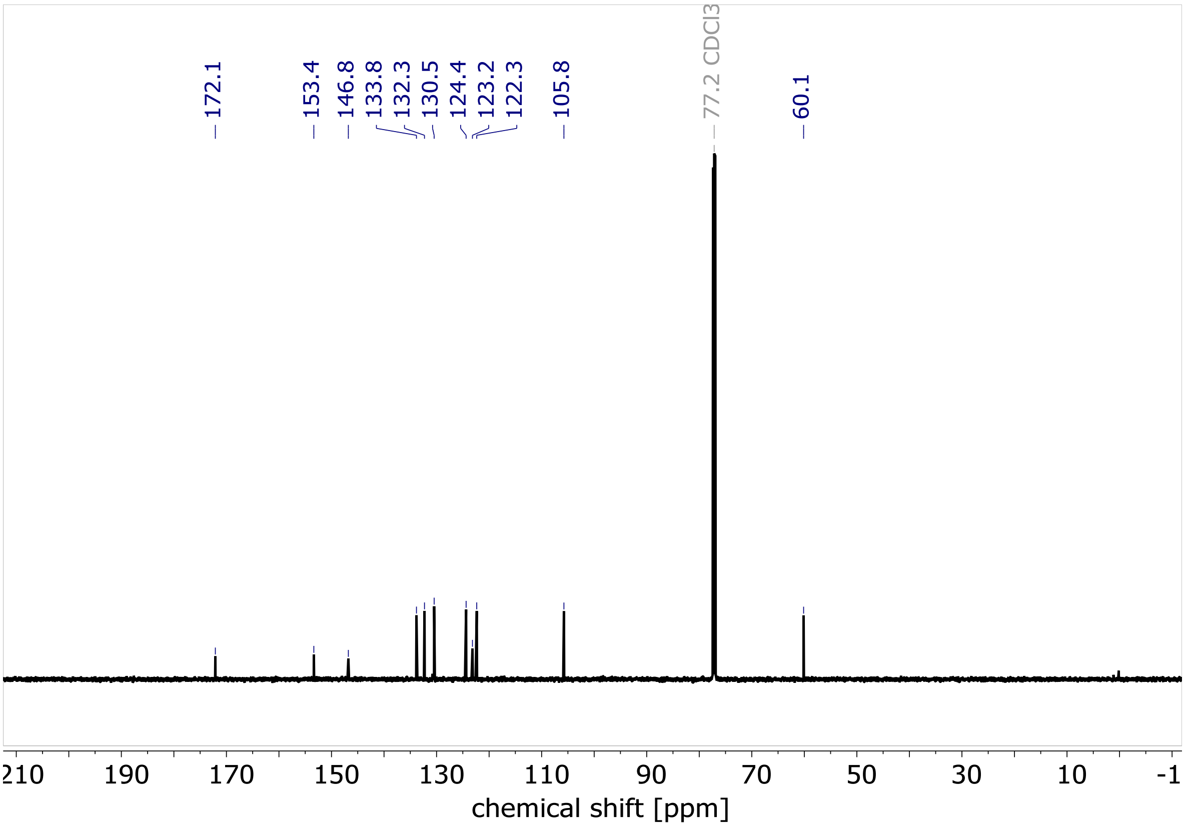

***Meta*-thiophenylazo-phenyl-*N*-Boc-hydrazide 8**

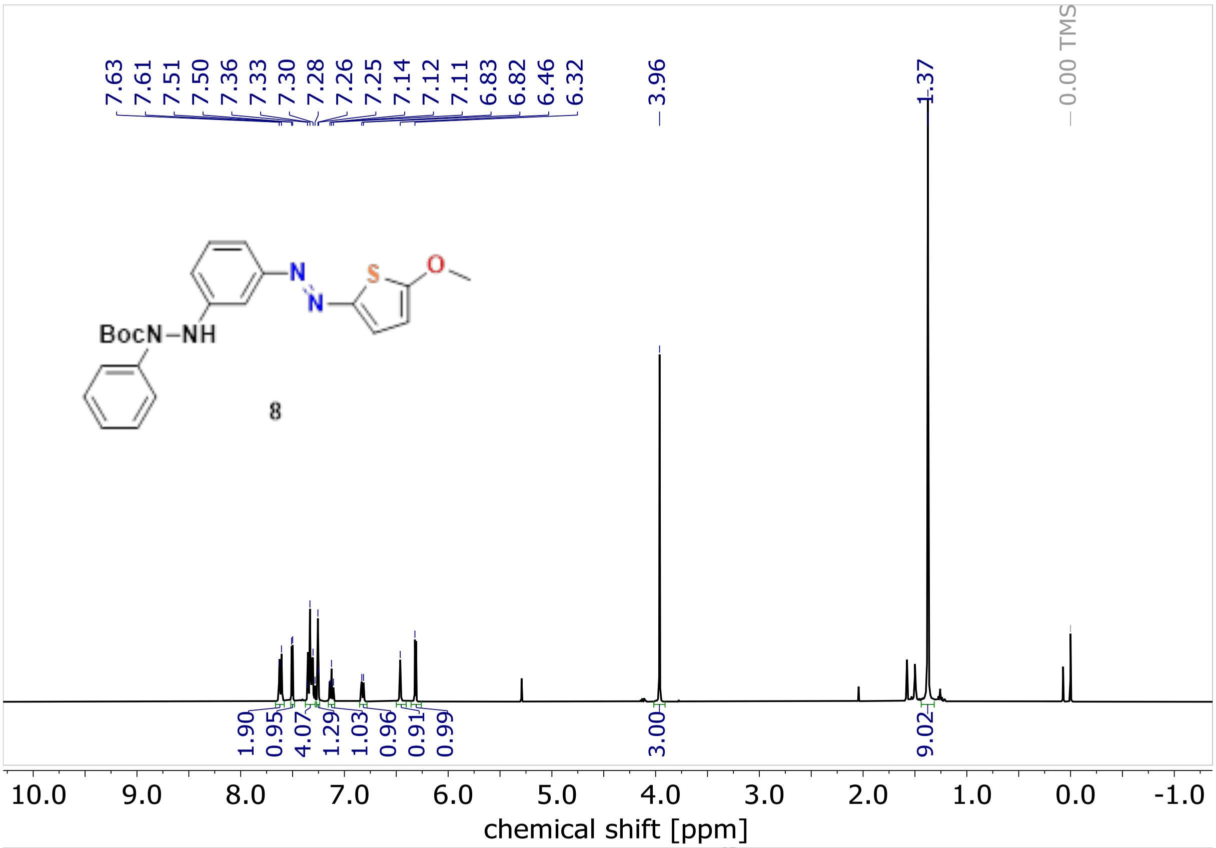

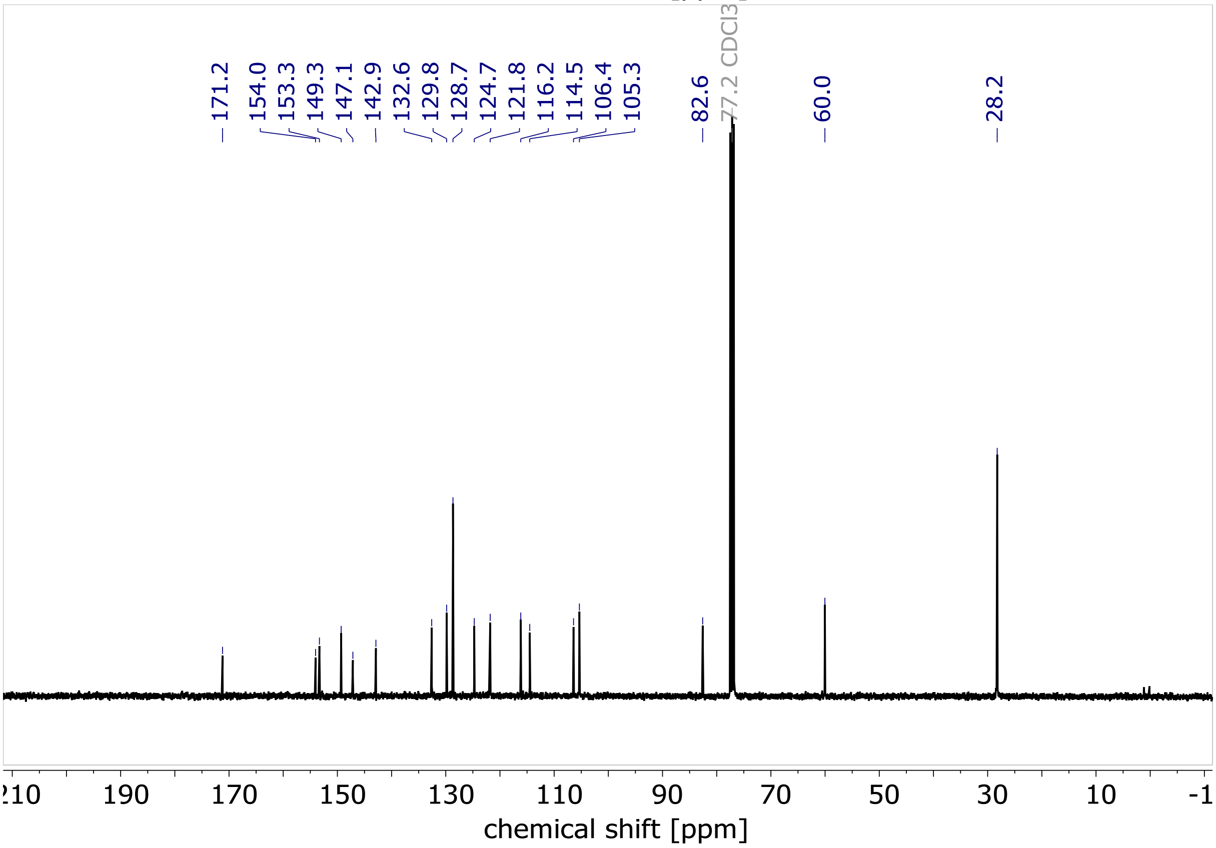

1-Phenyl-3-(5-methoxythiophenyl)bisazobenzene 4

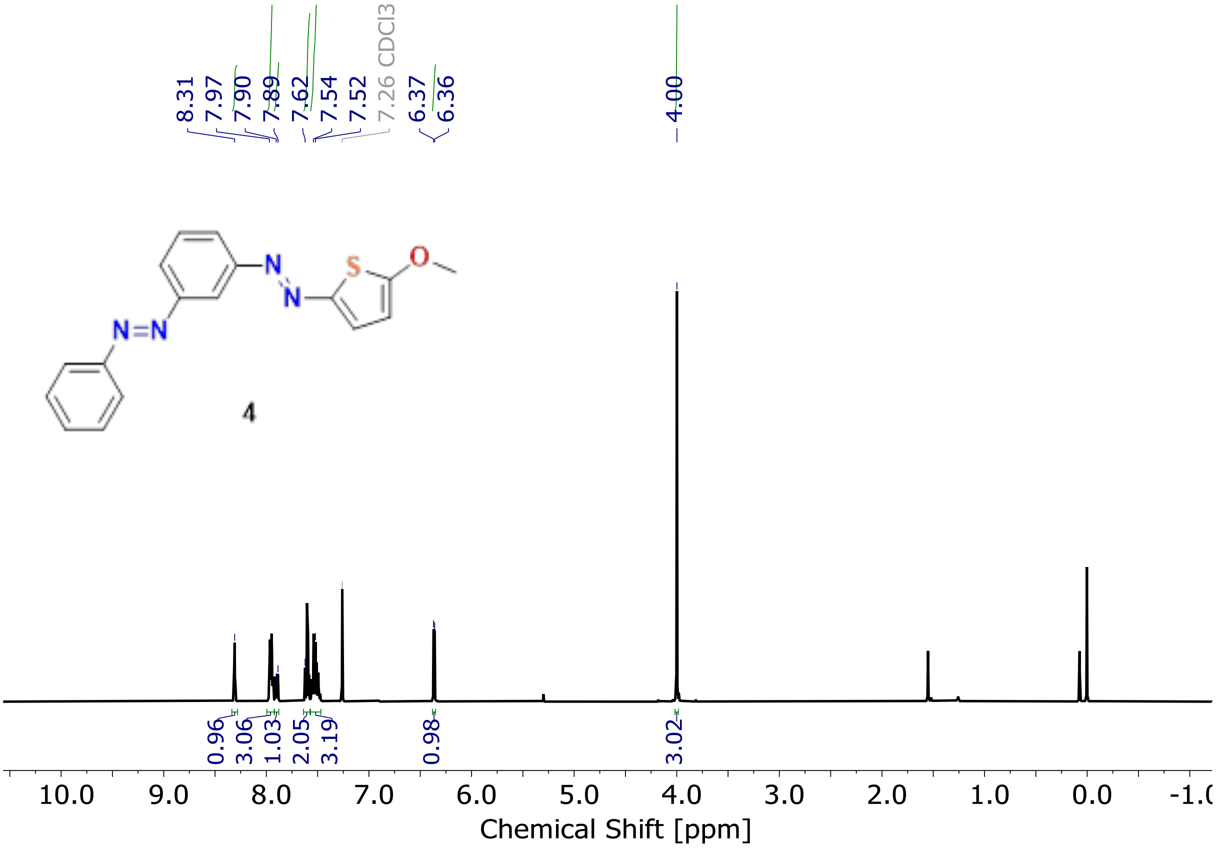

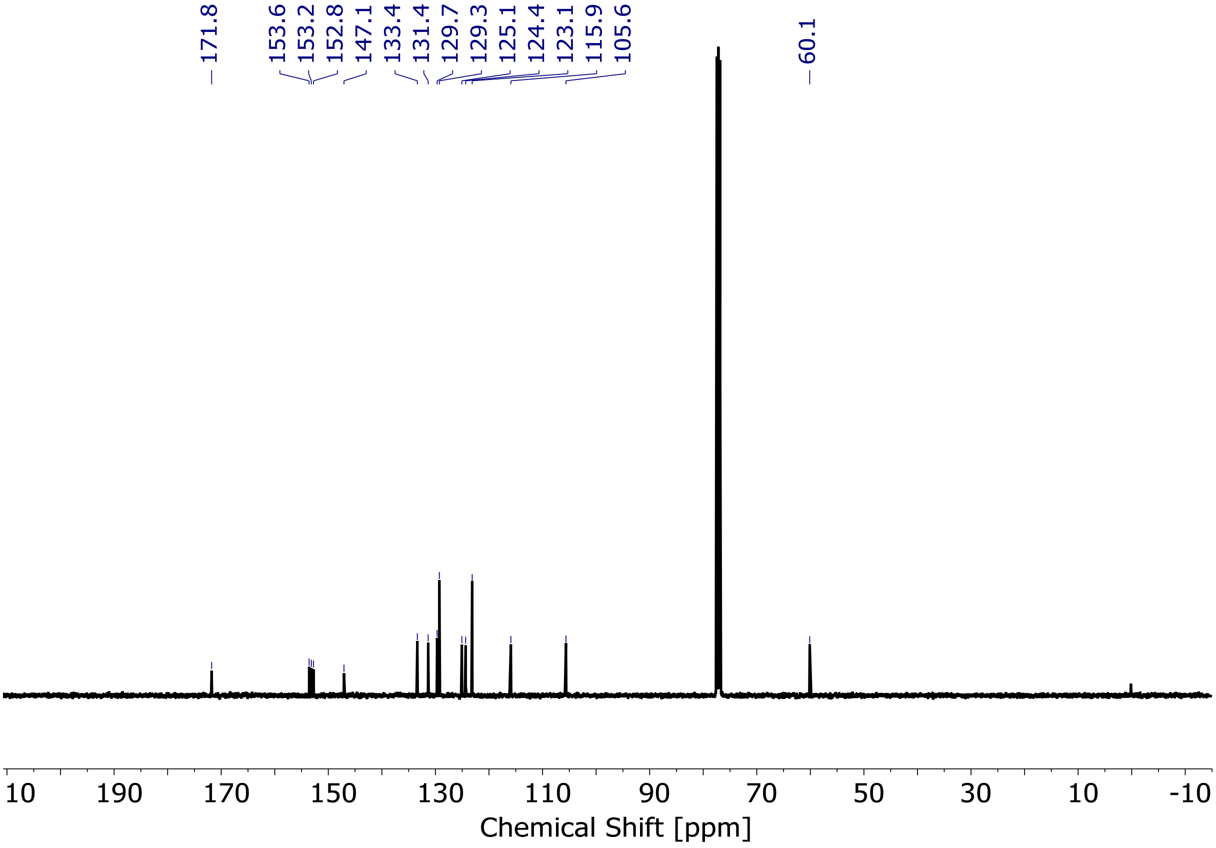

UV-Vis Spectroscopy

Solvents for UV/Vis spectroscopy were purchased from Merck (Uvasol® quality). The measurements were carried out with a SPECORD® 200 PLUS spectrophotometer equipped with two automatic eight-fold cell changers and a Peltier element thermostat system (0.1 °C accuracy) by Analytik Jena. The system was operated with the ASpect UV software by Analytik Jena. The sample solutions were measured in QS High Precision Cells made of Quartz Suprasil® with a light path of 10 mm by Hellma Analytics. Photoisomerization experiments were carried out in concentrations of $2 \cdot 10^{-5}$ mol/L. Stock solutions ($c_{stock} \sim 4 \cdot 10^{-4}$ mol/L) were prepared from freshy purified samples, and then diluted prior to every UV/Vis experiment.

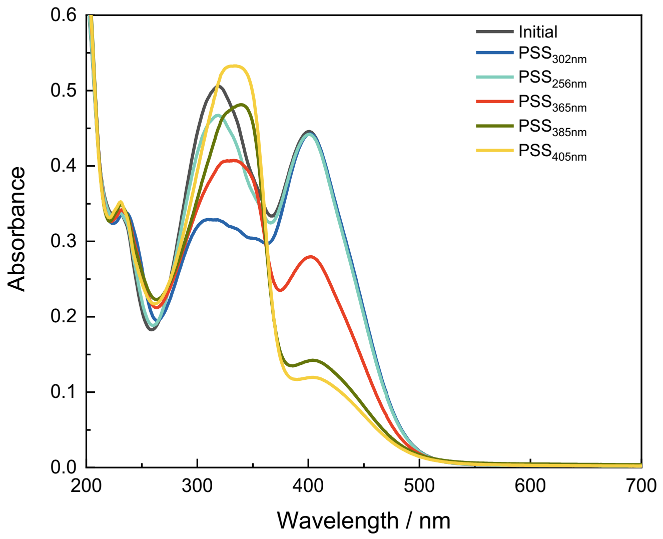

Figure S2: UV-Vis absorption spectra of **4** (2.0×10^{-5} M) in ACN. The pristine sample (black) was irradiated subsequently with 302 nm (blue), 256 nm (turquoise), 365 nm (red), 385 nm (green) and 405 nm (yellow) light irradiation to their respective photostationary state.

Compound **4** has to be stored under exclusion of light. By exposure to ambient conditions, the thiophenyl-part of the substance is switched from (E_{phenyl},E_{thio}) to (E_{phenyl},Z_{thio}). To demonstrate that, a solution of **4** in quartz cuvettes (c = 2·10^{-5} mol/L, ACN as well as DMSO) were simultaneously stored under ambient light and under light exclusion. There are no changes in the spectra when storing the solution under exclusion of light, but switching from (E_{phenyl},E_{thio}) to (E_{phenyl},Z_{thio}) under ambient light conditions was observed.

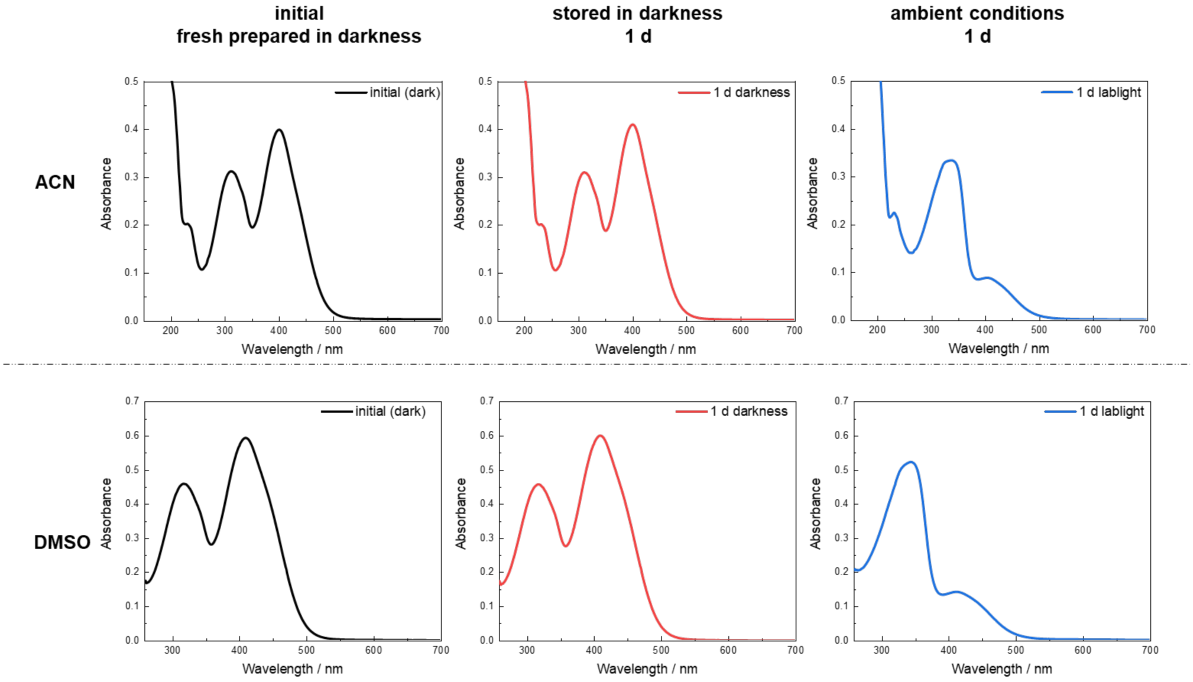

Figure S3: UV-Vis absorption spectra of **4** (2.0 x 10^{-5} M) in ACN and DMSO in its pristine state (black), after 1 d in the dark (red) and after 1 d at ambient light conditions (blue).

^{1}H-NMR Isomerization experiments

NMR experiments with in-situ sample irradiation

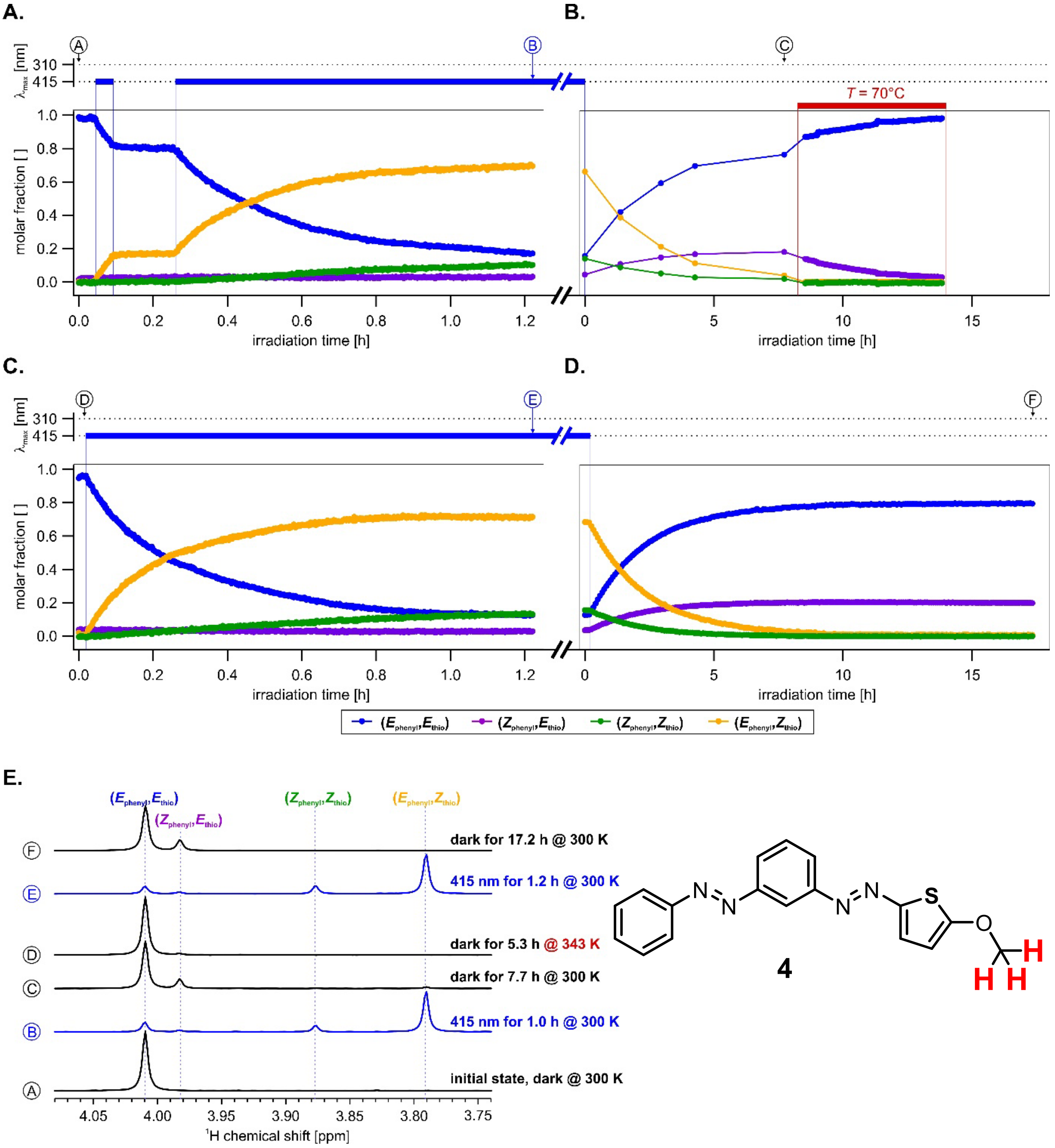

Figure S4: Photoisomerization of MeO-TphAB-AB compound **4** by illumination with 415 nm light and thermal relaxation, observed by ^{1}H-NMR. Irradiation with 415 nm was performed while the sample was located inside the NMR probe (in-situ). **A – D.** Change of the molar fractions of the (E_{phenyl},E_{thio}) (blue), the (Z_{phenyl},E_{thio}) (magenta), the (Z_{phenyl},Z_{thio}) (green) and the (E_{phenyl},Z_{thio}) (yellow) isomers as a function of irradiation time. Periods of irradiation with 415 nm are marked atop the graphs (blue bars). Experiments were performed at 300 K (27°C), with exemption of the time period marked in red, where temperature was raised to 343 K (70°C) for faster relaxation. **E.** ^{1}H-NMR spectra taken at points A – F, indicated at the top of panels A – D. Only the methoxy-region, highlighted in the chemical structure, is shown in the spectra for clarity.

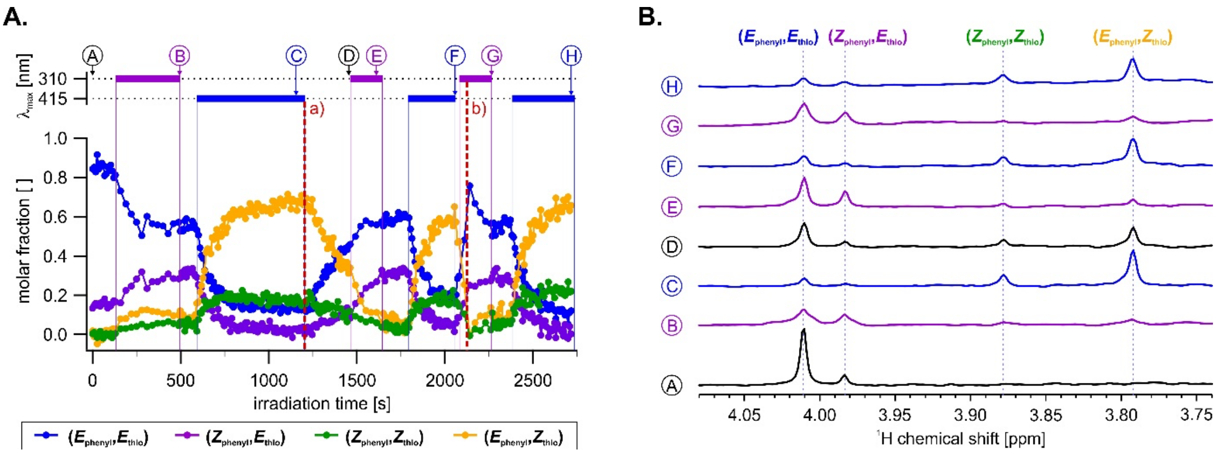

Figure S5: Photoisomerization of MeO-TphAB-AB compound **4** by alternating illumination with 310 nm and 415 nm light, observed by ^{1}H-NMR. Irradiation with 415 nm was performed while the sample was located inside the NMR probe (in-situ), whereas for irradiation with 310 nm, the sample was repeatedly removed from the NMR for illumination outside of the NMR (ex-situ). **A.** Change of the molar fractions of the (E_{phenyl},E_{thio}) (blue), the (Z_{phenyl},E_{thio}) (magenta), the (Z_{phenyl},Z_{thio}) (green) and the (E_{phenyl},Z_{thio}) (yellow) isomers as a function of irradiation time. The sequence of irradiation events is represented at the top of the graph (310 nm, magenta bar; 415 nm blue bar). Points in time where the spectra shown in panel B were recorded are indicated by markers A – H. At event „a)“ (indicated by a red dotted line), the LED power source failed. The component was replaced by an identical power source prior to the next irradiation period. At event „b)“ indicated by a red dotted line, the sample tube broke during manual sample shuttling. The sample was transferred to a new tube in the dark and the measurement was continued after reassembly of the setup. Sample transfer to the new tube, reassembly and reshimming of the setup took 21 minutes. This period was not added to the irradiation time stated. **B.** ^{1}H-NMR spectra taken at different point in time A – H during of the measurement, as indicated at the top of panel A. Only the methoxy-region of the spectra is shown for clarity.

Table S3: Molar fractions of the (E_{phenyl},E_{thio}), the (Z_{phenyl},E_{thio}) the (Z_{phenyl},Z_{thio}) and the (E_{phenyl},Z_{thio}) isomers of **4** at the timepoints marked in Figure S4 and Figure S5, as well as molar fractions in the photostationary states (PSS) obtained after irradiation with 415 nm or with 310 nm. A minimum error ε_{min} of 2% was assumed for each individual measurement of the molar fraction, and the error ε on the molar fraction in the PSS was estimated via $\varepsilon = \sqrt{(STD)^2 + (N-1)^{-1}(\varepsilon_{min})^2}$, where STD is the standard deviation of the individual measurements, and N is the number of individual measurements.

	TIMEPOINT	(E_{phenyl},E_{thio})	(Z_{phenyl},E_{thio})	(Z_{phenyl},Z_{thio})	(E_{phenyl},Z_{thio})
Figure S4	**A**	99%	1%	0%	0%
	**B**	17%	3%	10%	70%
	**C**	76%	18%	2%	4%
	**D**	96%	4%	0%	0%
	**E**	13%	4%	16%	67%
	**F**	80%	20%	0%	0%
Figure S5	**A**	84%	13%	0%	2%
	**B**	51%	31%	7%	11%
	**C**	12%	2%	15%	71%
	**D**	44%	11%	11%	34%
	**E**	56%	27%	6%	11%
	**F**	11%	4%	22%	63%
	**G**	57%	27%	2%	14%
	**H**	11%	4%	22%	63%
**PSS**	**PSS (415 nm)**	(13 ± 3)%	(3 ± 2)%	(17 ± 6)%	(67 ± 4)%
	**PSS (310 nm)**	(55 ± 4)%	(28 ± 3)%	(5 ± 3)%	(12 ± 2)%

Fitting of thermal relaxation data

To extract thermal half-life times in **4**, the molar fractions from the thermal relaxation data shown in Figure S4D (collected at 300 K) were initially fitted monoexponentially under the assumption that the relaxation processes of the two photochromic groups are independent and that the relaxation of the azobenzene is negligible over the duration of the experiment. This led to discrepancies between the different values obtained from the different isomers.

Thus a more sophisticated analysis was performed. The thermal relaxation data shown in Figure S4D (collected at 300 K) fitted in two different approaches: **Fit A** assumes fully independent relaxation of both photoswitchable moieties (**scenario A**), whereas **fit B** assumes a scenario in which the configuration of one of the photoswitchable moieties can impact the thermal relaxation rate of the other moiety (**scenario B**). In both cases, simultaneous fitting of all four molar fractions shown was performed, to extract the thermal relaxation rates for the 5-methoxythiophenyl-azobenzenyl moiety at 300 K (see Table S4).

Fit A, which assumes fully independent switching, fits the data in Figure S4D (collected at 300 K) well, with fitting residuals showing small, but systematic deviations from the experimental data (see **Figure S6**). These are in a similar range (-1.5% to +1%) to the error that should be assumed for the accuracy of the molar fraction measurement (±2%; estimated from the partial signal overlap occurring between the H_{23} signals of E_{phenyl},E_{thio} and Z_{phenyl},E_{thio}). The thermal half-life time for $Z_{thio} \rightarrow E_{thio}$ relaxation at 300 K, assuming independent switching, is (1.54 ± 0.01) h (see Table S4).

Fitting the same data with the model that considers possible dependencies of the thermal relaxation rates on the configuration of the other photoswitchable moiety (**Fit B**), shows that the small systematic deviations observed in fit A do not occur in the fitting residuals of fit B, but that random scatter is present.

Indeed, the fitted rate constants for thermal relaxation of the thiol moiety differ outside of the error range estimated ($\tau_{1/2}$(E_{phenyl},Z_{thio} $\rightarrow$ E_{phenyl},E_{thio}) = (1.58 ± 0.01) h; $\tau_{1/2}$(Z_{phenyl},Z_{thio} $\rightarrow$ Z_{phenyl},E_{thio}) = (1.21 ± 0.03) h) (see Table S4).

Within the experimental errors that need to be assumed, the thermal relaxation data shown in Figure S4 therefore is consistent with both, fully independent thermal relaxation of the two photoswitchable moieties as well as dependent relaxation in **4**.

Fitting of the data collected at 70°C, shown in Figure S4B is only possible under the assumption of fully independent relaxation (**scenario A**), since thermal relaxation of the 5-methoxythiophenylazobenzenyl moiety proceeds too fast at this temperature and errors are of the same order of magnitude as the value itself. Thus, these values should be viewed with caution.

In summary, irrespective of the model chosen the half-life time for thermal relaxation at 300K for (E_{phenyl},Z_{thio} $\rightarrow$ E_{phenyl},E_{thio}) is ca 1.5 h. In model B the value for starting from the (Z_{phenyl}) is slightly less (1.2 h).

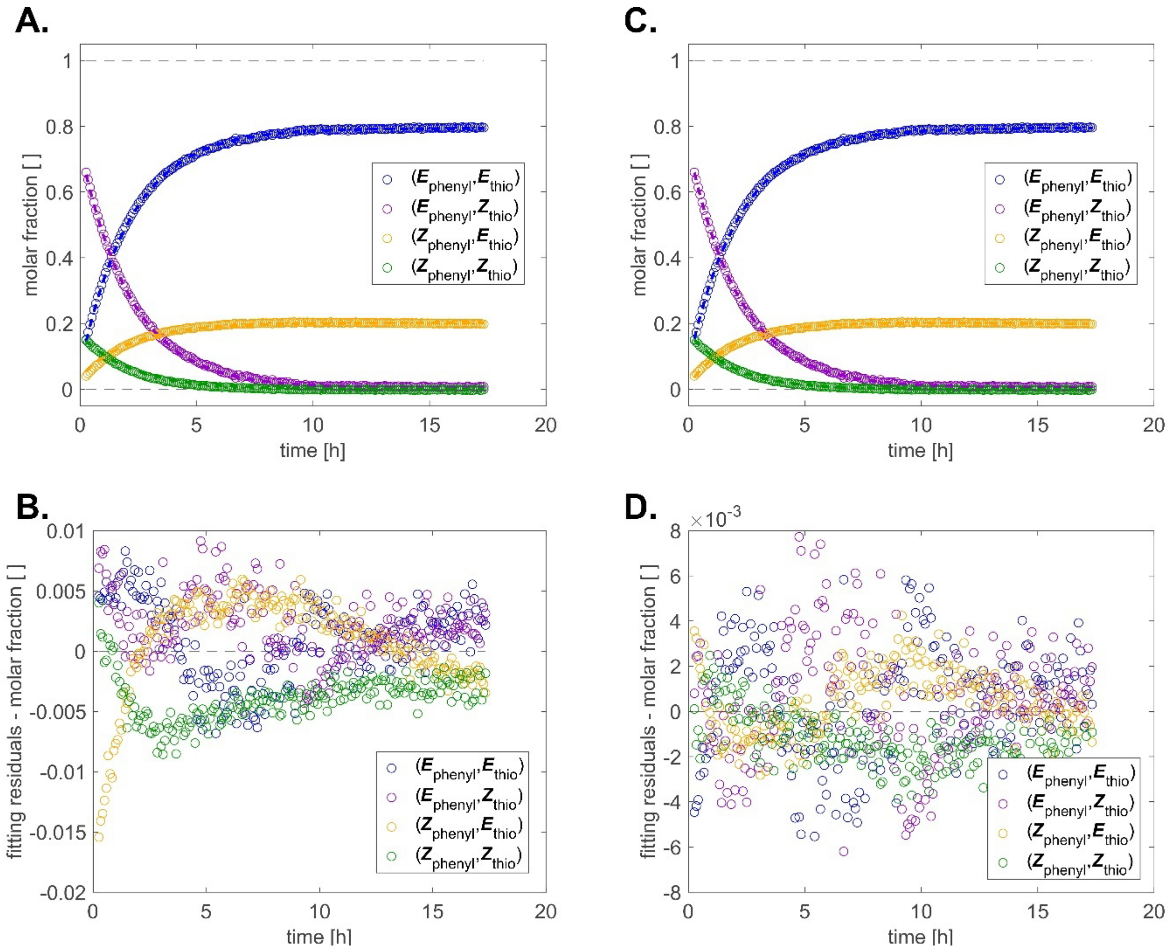

Figure S6: Fits of the thermal relaxation data at 300 K, shown in Figure S4. **A.** & **B.**: Fit and fitting residuals for a fit according to scenario A, which assumes fully independent relaxation of the photoswitchable units. **C.** & **D.**) Fit and fitting residuals for a fit according to scenario B, in which a possible dependence of the thermal relaxation rates of the photoswitchable units on the configuration of the other photoswitchable unit is included in the model.

Fitting procedure

Fitting of the thermal relaxation data was performed, assuming the first-order kinetics scheme shown in Scheme 1. To describe the dynamics of the system during thermal relaxation, we use

$$\frac{d}{dt}\begin{pmatrix} x(E_{phenyl}, E_{thio}) \\ x(E_{phenyl}, Z_{thio}) \\ x(Z_{phenyl}, E_{thio}) \\ x(Z_{phenyl}, Z_{thio}) \end{pmatrix} = \frac{d}{dt}\begin{pmatrix} x_A \\ x_B \\ x_C \\ x_D \end{pmatrix} = \boldsymbol{K}\begin{pmatrix} x_A \\ x_B \\ x_C \\ x_D \end{pmatrix} \quad (1)$$

with

$$\boldsymbol{K} = \begin{pmatrix} -(k_{AB}+k_{AC}+k_{AD}) & k_{BA} & k_{CA} & k_{DA} \\ k_{AB} & -(k_{BA}+k_{BC}+k_{BD}) & k_{CB} & k_{DB} \\ k_{AC} & k_{BC} & -(k_{CA}+k_{CB}+k_{CD}) & k_{DC} \\ k_{AD} & k_{BD} & k_{CD} & -(k_{DA}+k_{DB}+k_{DC}) \end{pmatrix}. \quad (2)$$

For simplicity, we herein abbreviated $(E_{\text{phenyl}}, E_{\text{thio}})$ as A, $(E_{\text{phenyl}}, Z_{\text{thio}})$ as B, $(Z_{\text{phenyl}}, E_{\text{thio}})$ as C and $(Z_{\text{phenyl}}, Z_{\text{thio}})$ as D. Time evolution of the molar fractions from their initial values $x_{k,0}$ was computed via

$$\begin{pmatrix} x_A(t) \\ x_B(t) \\ x_C(t) \\ x_D(t) \end{pmatrix} = e^{\boldsymbol{K}t}\begin{pmatrix} x_{A,0} \\ x_{B,0} \\ x_{C,0} \\ x_{D,0} \end{pmatrix}, \quad (3)$$

Where $e^{\boldsymbol{K}t}$ is the matrix exponential of $\boldsymbol{K}t$, with t as the time.

Least squares fitting of the thermal relaxation data with equation 3**Error! Reference source not found.** was performed in MATLAB® R2024b.[30] The sum of the squared deviations between all four traces of experimental data to computed data was minimized. During fitting, the sum of all molar fractions was constrained to $\sum_k x_k = 1$.
For fitting, two scenarios were considered: **Scenario A** assumes fully independent switching of both photoswitchable moieties, whereas **scenario B** assumes a scenario in which the configuration of one of the photoswitchable moieties can impact the thermal relaxation rate of the other. In both scenarios, processes of simultaneous switching of both switchable moieties (e.g. direct interconversion from $E_{\text{phenyl}}, E_{\text{thio}}$ to $Z_{\text{phenyl}}, Z_{\text{thio}}$ via k_{AD}) were excluded from the model (in all cases: $k_{AD} = k_{BC} = k_{CB} = k_{DA} = 0$). The independent switching assumed in scenario A further enforces $k_{AB} = k_{CD}$, $k_{AC} = k_{BD}$, $k_{BA} = k_{DC}$ and $k_{CA} = k_{DB}$. Rate constants were converted to half-life times for presentation in Table S4, according to $\tau_{1/2,i} = \ln(2)/k_i$.

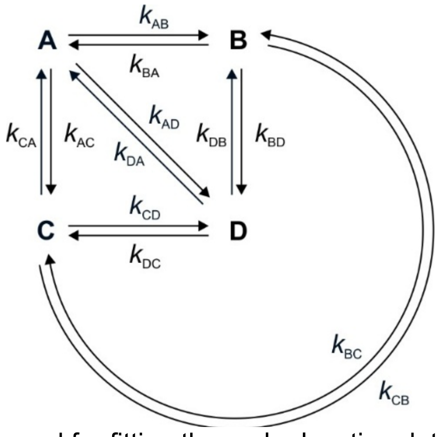

Scheme 1: Kinetics scheme assumed for fitting thermal relaxation data.

Table S4: Thermal relaxation rates of the (E_{phenyl},E_{thio}), the (Z_{phenyl},E_{thio}) the (Z_{phenyl},Z_{thio}) and the (E_{phenyl},Z_{thio}) isomers of **4**, extracted from the data shown in Figure S4 B & D. Data was measured at 300 K (27°C) or at 343 K (70°C) as indicated, using 5 mM **4** in DMSO-d$_6$. Thermal relaxation rates were extracted by simultaneously fitting equation 3**Error! Reference source not found.** to the molar fractions of all four isomers of **4**. For Figure S4 D, the fit was performed over all data collected after turning off the illumination. For Figure S4 B, the fitting range was restricted to the data measured at 343 K (70°C) (8.5 – 12.7 h). For convenience, the first-order reaction rates, as well as the half-life times, computed via $\tau_{1/2,i} = \ln(2)/k_i$ are shown. Values discussed in the text are highlighted in bold. Note, that a value of 0 indicates that thermal relaxation is not detectable over the time of the experiment.

	**PROCESS**		***SCENARIO A* (INDEPENDENT RELAXATION)**		***SCENARIO B* (DEPENDENT RELAXATION)**	
			***k* [h^{-1}]**	**$\tau_{1/2}$ [h]**	***k* [h^{-1}]**	**$\tau_{1/2}$ [h]**
**FIGURE S4 D (300 K)**	**E_{phenyl},Z_{thio} → E_{phenyl},E_{thio}**	k_{BA}	0.450 ± 0.003	**1.539 ± 0.009**	0.438 ± 0.003	**1.582 ± 0.009**
	**Z_{phenyl},Z_{thio} → Z_{phenyl},E_{thio}**	k_{DC}	$k_{DC} = k_{BA}$	$\tau_{1/2,DC} = \tau_{1/2,BA}$	0.575 ± 0.012	**1.21 ± 0.03**
	**Z_{phenyl},E_{thio} → E_{phenyl},E_{thio}**	k_{CA}	0.00 ± 0.07	a)	0.00 ± 0.06	a)
	**Z_{phenyl},Z_{thio} → E_{phenyl},Z_{thio}**	k_{DB}	$k_{DB} = k_{CA}$	$\tau_{1/2,DB} = \tau_{1/2,CA}$	0.00 ± 0.13	a)
FIGURE S4 B (70°C)	E_{phenyl},Z_{thio} → E_{phenyl},E_{thio}	k_{BA}	b)	b)	b)	b)
	Z_{phenyl},Z_{thio} → Z_{phenyl},E_{thio}	k_{DC}	$k_{DC} = k_{BA}$	$\tau_{1/2,DC} = \tau_{1/2,BA}$	b)	b)
	Z_{phenyl},E_{thio} → E_{phenyl},E_{thio}	k_{CA}	0.34 ± 0.03	2.1 ± 0.2	b)	b)
	Z_{phenyl},Z_{thio} → E_{phenyl},Z_{thio}	k_{DB}	$k_{DB} = k_{CA}$	$\tau_{1/2,DB} = \tau_{1/2,CA}$	b)	b)

a) Presentation of half-life times not reasonable (division by zero).
b) Extraction from experimental data not possible.

^{1}H-NMR signal assignment

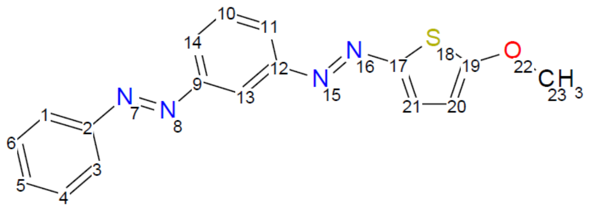

Table S5: ^{1}H-NMR signal assignment for the four isomers of MeO-TphAB-AB compound **4**. For fields with multiple entries, no specific assignment of the resonances to the sites listed is given. All data was collected in DMSO-d6. Chemical shifts are reported in ppm and are referenced against TMS (0.0 ppm).

POSITIONS	($E_{phenyl}E_{thio}$)	(Z_{phenyl},E_{thio})	(Z_{phenyl},Z_{thio})	(E_{phenyl},Z_{thio})
H23	**4.03**	**4.00**	**3.89**	**3.80**
**H21**	7.86	7.75	7.88	7.98
H20	6.65	6.61	6.50	6.53
H14 & H11	7.99, 7.92	b)	6.73, 6.64	7.12, 7.92
H13	8.12	b)	6.46	7.34
H10	7.76	b)	7.40	7.77
H5, H4 **&** H3	7.95, 7.63, 7.63	b)	6.90, 7.30, a)	7.92, 7.61, a)

a) No assignment. **b)** Signals observed at 6.82, 6.90, 7.17, 7.32, 7.41 & 7.49 ppm can be assigned to the (Z_{phenyl},E_{thio}) isomer, but the data available does allow the assignment to which of the sites listed these signals belong. COSY and NOESY data, DOI: 10.5281/zenodo.16222847.

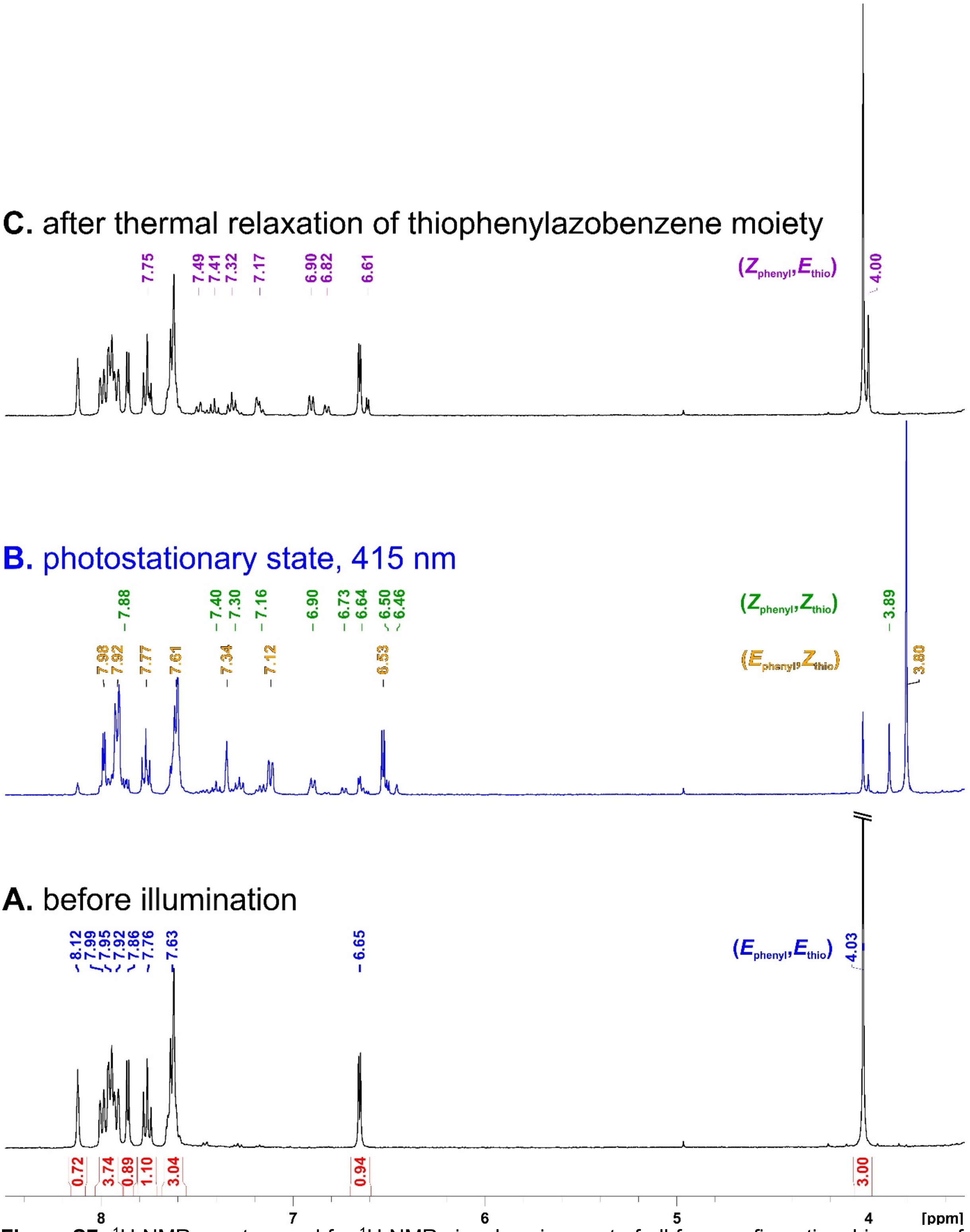

Figure S7: ^{1}H-NMR spectra used for ^{1}H-NMR signal assignment of all four configurational isomers of MeO-TphAB-AB compound **4**. For signal assignment in $CDCl_3$, please consult data presented on pages S9 and S12. **A.** Spectrum collected prior to exposing the sample to light (right before timepoint "A" in panel A of Fig S4). **B.** Spectrum collected in the PSS obtained by continuous irradiation with 415 nm light (data point at 0 hours in panel B of Fig S4). **C.** Spectrum collected after thermal relaxation of the thiophenylazobenzene moiety from the PSS obtained with 425 nm irradiation, but with incomplete thermal relaxation of the azobenzene moiety (timepoint "F" in panel D of Fig S4). All data collected at 300 K. All chemical shifts referenced against TMS (δ(TMS) = 0.00 ppm).

Computational Methods

All computations were performed using QChem 5.2[31] or Orca 5.0.1.[32] All geometries were optimized at DFT level using the CAM-B3LYP/def2-TZVP exchange-correlation functional/basis set combination and the D3(BJ) dispersion correction.[33-35] Subsequent frequency calculations were undertaken to verify the obtained equilibrium geometries as minima on the potential energy surface. The ten energetically lowest singlet excited states were computed using linear-response time-dependent DFT (TD-DFT) with the same functional/basis set combination. At the same level of theory, constrained potential energy surface scans of the lowest lying excited state along the CNNC dihedral angle rotations were computed. Unconstrained excited state geometry optimizations of the third, fourth and fifth excited state employed the state-following algorithm implemented in Orca. Detachment and attachment density plots are used for the characterization of the excited states. The detachment densities correspond to that part of the density that is removed upon excitation and rearranged as attachment density.[32] Detachment and attachment densities were computed at the ground state equilibrium geometry.

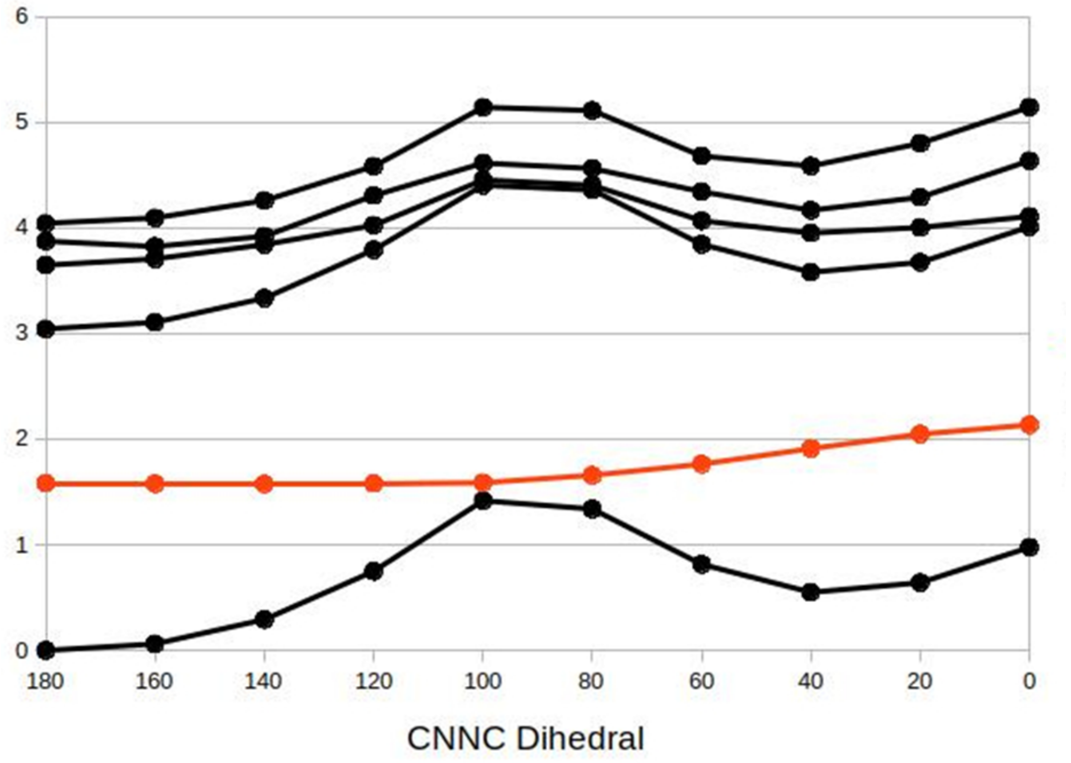

Figure S8: Relaxed potential energy surface scan of the relaxed nπ* state located at the AB branch along the corresponding CNNC dihedral angle. The red line corresponds to the optimized state.

XYZ-coordinates:

(*E*,*E*)-**4**:

37

E = -1348.47177770719

C	0.9412595796	0.7287120014	-0.0109653555
C	0.9447173422	2.1186676903	-0.0011113475
C	-0.2608223097	2.7967582633	0.0060869142
C	-1.4628848547	2.1119418490	0.0048971648
C	-0.2582936669	0.0362496702	-0.0149114846
C	-1.4624399890	0.7211996938	-0.0052945730
H	-0.2559874285	-1.0447401381	-0.0243374342
H	-2.4058032564	2.6383000073	0.0111076072
H	-0.2656815497	3.8786763139	0.0126444921
H	1.8867977373	2.6457695306	0.0000112980
N	-2.6296840946	-0.0776895097	-0.0056162346
N	-3.7006166485	0.5554259064	-0.0097325107
C	-4.8471976778	-0.1864016197	-0.0070928018
C	-6.1057243982	0.3447775923	-0.0132366707
C	-6.6178069950	-1.8916507546	0.0040825099
C	-7.1240375262	-0.6227426782	-0.0072407295
H	-6.2703951570	1.4113748119	-0.0217306324
H	-8.1836251588	-0.4212459380	-0.0103063545
S	-4.8876896768	-1.9336952031	0.0065471873
N	2.1045839439	-0.0786588673	-0.0219379627
N	3.1766211149	0.5311891828	0.0308568944
C	4.3347238244	-0.2866912019	0.0135112665
C	5.5444003588	0.3813982956	0.1135094840
C	6.7307145855	-0.3308322135	0.1056533575
C	6.7045348327	-1.7097256636	-0.0055854319
C	5.4904228299	-2.3769863357	-0.1077181407
C	4.3046882760	-1.6733104361	-0.0985486618
H	7.6299588962	-2.2700741576	-0.0138845602

H	5.4739196897	-3.4553508510	-0.1958127758
H	3.3515101548	-2.1748213964	-0.1773146029
H	5.5317599993	1.4594692185	0.1979130680
H	7.6748122515	0.1907627879	0.1855500136
O	-7.3781696046	-2.9877276364	0.0124997359
C	-6.7383891340	-4.2529099026	0.0276627654
H	-7.5318264184	-4.9939052385	0.0342023040
H	-6.1229331405	-4.3652971743	0.9226891589
H	-6.1193267314	-4.3849658987	-0.8621769568

(Z_{phenyl},E_{thio}) **-4**:

37

E = -1348.45052515499

C	1.0685750684	0.8609002287	0.6302308665
C	1.0759768270	2.2418635909	0.7259135171
C	-0.1283393332	2.9289128032	0.7044067333
C	-1.3240410924	2.2562075788	0.5651639614
C	-0.1257794633	0.1694130761	0.5480501212
C	-1.3200546338	0.8665209699	0.4766592827
H	-0.1398017988	-0.9112151967	0.5042024180
H	-2.2642157288	2.7849506663	0.5254417290
H	-0.1268166489	4.0076069541	0.7860480470
H	2.0129345261	2.7728783512	0.8159428936
N	-2.4814504302	0.0778230792	0.3028809214
N	-3.5303176717	0.7235251132	0.1249012576
C	-4.6695583068	-0.0018388893	-0.0717137387
C	-5.8996587188	0.5487732040	-0.2975331978
C	-6.4339554974	-1.6793884228	-0.4105164425
C	-6.9141034626	-0.4025291539	-0.4913608316
H	-6.0456451198	1.6178666929	-0.3224575162
H	-7.9525273179	-0.1849673949	-0.6855165183
S	-4.7342773700	-1.7486328605	-0.0923636769

N	2.2865663912	0.1116066088	0.7150423149
N	3.1278962718	0.1713996139	-0.1811602249
C	2.8791771979	0.8992209615	-1.3902258428
C	3.7639761738	1.9068826727	-1.7357362013
C	3.5874528891	2.5959707036	-2.9216662681
C	2.5604185776	2.2492930967	-3.7840558823
C	1.7094940078	1.2076715424	-3.4550848848
C	1.8612368440	0.5321142166	-2.2583991579
H	2.4314404546	2.7812738635	-4.7169054578
H	0.9164301438	0.9197814606	-4.1323493413
H	1.1948331514	-0.2788206171	-2.0022055047
H	4.5775685473	2.1490998569	-1.0652148025
H	4.2644467978	3.3991946465	-3.1801454365
O	-7.1963858002	-2.7630886298	-0.5605155405
C	-6.5792393071	-4.0378549198	-0.4916909025
H	-7.3693712637	-4.7657712936	-0.6477261943
H	-6.1224606680	-4.1954147012	0.4875136596
H	-5.8220542359	-4.1437294725	-1.2713241593

(E_{phenyl},Z_{thio})-**4**:

37

E = -1348.45059423285

C	1.1705478682	1.1940187415	0.1097969872
C	1.3432503003	2.5680081149	-0.0168315056
C	0.2416027678	3.3938266093	0.0905962541
C	-1.0212619776	2.8705516512	0.3258755738
C	-0.0890538353	0.6612198377	0.3222636195
C	-1.1881048946	1.4984262871	0.4262724933
H	-0.2003588501	-0.4103200112	0.4175537852
H	-1.8812953843	3.5184835153	0.4287283272
H	0.3622871938	4.4650233086	-0.0001931520
H	2.3328021553	2.9651498465	-0.1846849966
N	-2.4526629187	0.9494026570	0.7847238413
N	-3.2335425200	0.4831229213	-0.0556261358

C	-3.0698198303	0.4589323446	-1.4264477970
C	-4.0294233526	-0.1327357755	-2.2039932993
C	-2.6374332353	0.6117549969	-3.8602020958
C	-3.7943961589	-0.0605001550	-3.5820756709
H	-4.8914507351	-0.5999326576	-1.7517215384
H	-4.4344600787	-0.4635209614	-4.3510826379
S	-1.8115353871	1.1450470126	-2.4422298585
N	2.2116100583	0.2374807213	0.0340220230
N	3.3595285846	0.6897234462	0.0704856490
C	4.3841294018	-0.2879586567	-0.0099157105
C	5.6667394392	0.1524621340	0.2733603400
C	6.7260425152	-0.7373284742	0.2367343421
C	6.5026662979	-2.0610321003	-0.0988112554
C	5.2177833632	-2.4965553871	-0.3962973407
C	4.1561651871	-1.6175464378	-0.3507918444
H	7.3295128122	-2.7579443461	-0.1329979408
H	5.0473720556	-3.5309501626	-0.6642866605
H	3.1498044862	-1.9394709324	-0.5743522628
H	5.8102433240	1.1931192373	0.5300599411
H	7.7259362565	-0.3968379612	0.4691393886
O	-2.1902602533	0.8328724205	-5.0962357454
C	-0.9978382258	1.5846948360	-5.2532272081
H	-0.8193991082	1.6513247178	-6.3218844078
H	-0.1571142669	1.0828865412	-4.7698578753
H	-1.1157830547	2.5869121199	-4.8360256259

(Z_{phenyl},Z_{thio}) -**4**:

37

E = -1348.43084857808

C	1.1792835243	0.6534302571	0.1728570636
C	1.3869899119	2.0257117093	0.1915253384
C	0.2955172234	2.8704615069	0.2804134101
C	-0.9904314474	2.3645933168	0.3345430676
C	-0.0996831627	0.1343667609	0.2714368175

C	-1.1867532280	0.9898071093	0.3171091838
H	-0.2442099815	-0.9358935726	0.3323375178
H	-1.8442576563	3.0231387483	0.4162413868
H	0.4492743594	3.9404102537	0.3152604139
H	2.3891853830	2.4253503567	0.1572000408
N	-2.4790746376	0.4445106763	0.5575375282
N	-3.3427532989	0.3404399199	-0.3215420017
C	-3.2310662054	0.6652862003	-1.6587696046
C	-4.3255096079	0.5161045725	-2.4670104295
C	-2.8323592926	1.2632550854	-4.0298690962
C	-4.1167112075	0.8564632345	-3.8084298392
H	-5.2608243341	0.1596958382	-2.0621505604
H	-4.8575758270	0.8128807744	-4.5912952099
S	-1.8623448468	1.2433032269	-2.5997323725
N	2.2465012011	-0.2973501002	0.2318550131
N	3.2024307285	-0.2800251238	-0.5413301570
C	3.2753970367	0.6109733683	-1.6557308498
C	4.4228643170	1.3773046956	-1.7816015161
C	4.5758587958	2.2102240115	-2.8739400789
C	3.6110211691	2.2368036829	-3.8674941718
C	2.4921822992	1.4278896392	-3.7599698669
C	2.3119872850	0.6186324724	-2.6538316693
H	3.7396495697	2.8736416986	-4.7321506894
H	1.7522052221	1.4273065031	-4.5481501366
H	1.4321418859	-0.0037228830	-2.5646409683
H	5.1811288743	1.3163100492	-1.0124095696
H	5.4603640843	2.8270909119	-2.9585332653
O	-2.3842303120	1.6571622964	-5.2222383158
C	-1.0077723637	1.9614223582	-5.3562290671
H	-0.8696999196	2.3086348406	-6.3756310856
H	-0.4026627607	1.0687330891	-5.1863337437
H	-0.7102927812	2.7464825154	-4.6575425165

Ultrafast Time-resolved Spectroscopy

The transient absorption measurements were conducted on a home-built pump-probe system powered by Ti: sapphire laser offered by Spectra Physics (Spitfire Ace-100F-1K; Mai Tai SP-NSI; Empower 45, Spectra-Physics), which provides the fundamental wavelength (FW) centered at 800 nm with 100 fs full-width-half-maximum (FWHM) and 1 kHz repetition rate. A fraction of the FW is used to both seed and pump a self-made nonlinear optical parametric amplification, which is later up-converted into near-UV region by sum-frequency-generation in a BBO crystal. The 3 involved pumping wavelengths in this work (305 nm, 340 nm, and 420 nm) were all generated by this method. All the pulses which are applied to excite the samples possess FWHMs of 3-6 nm, to ensure the selectivity of the orthogonal switching, while the durations of the pulses had the FWHM from 50 fs to 60 fs as a trade-off. The probing pulses were generated from the same FW by focusing into a CaF2 crystal, which provide stable probing wavelengths from 315 nm to 650 nm.

The samples were prepared in a 1 mm quartz cuvette in both acetonitrile and Dimethyl sulfoxide (DMSO). The concentrations were adjusted to 0.7 OD for the peak of maximum absorption within the probing range. The excitation laser pulses (305nm, 340 nm and 420 nm) had power in the range of 30-70 nJ. For the experiments for Z->E isomerization of MeO-ThpAB **10** and MeO-ThpAB-AB **4**, the cuvette was exposed to 420 nm LED (M420L3, Thorlabs) to reach the photo stationary state during the pump-probe measurements. The difference spectra were measured by spectrometer provided by Avantas (AvaSpec-ULS2048CL-EVO-RS).

Ultrafast Time-resolved Measurements in DMSO

All the time-resolved transient absorption experiments were conducted separately in DMSO solvent with the same condition as described in the main text. Upon irradiation, the DMSO showed overwhelming solvent artefact compared to the difference absorption signal from the excited sample (Figure S9). Since the absorption spectrum of DMSO could extend up to 350 nm, 305 nm pulses create a broad and intensive solvent artefact (Figure S9, left), which is up to 8-fold of the ESA signal of azobenzene below 1 ps. Hence, the artefacts were cut off before 0.5 ps for all the transient data

excited by 305 nm pulse, neither were they included in the following lifetime analysis. For 340 nm and 400 nm excitation, the solvent artefacts of DMSO are less significant and were included in our data analysis routine. However, there were still difficulties as the artefacts are still much stronger than the signal and for 400 nm excitation, DMSO presents a light Raman band at 478 nm (Figure S9, right).

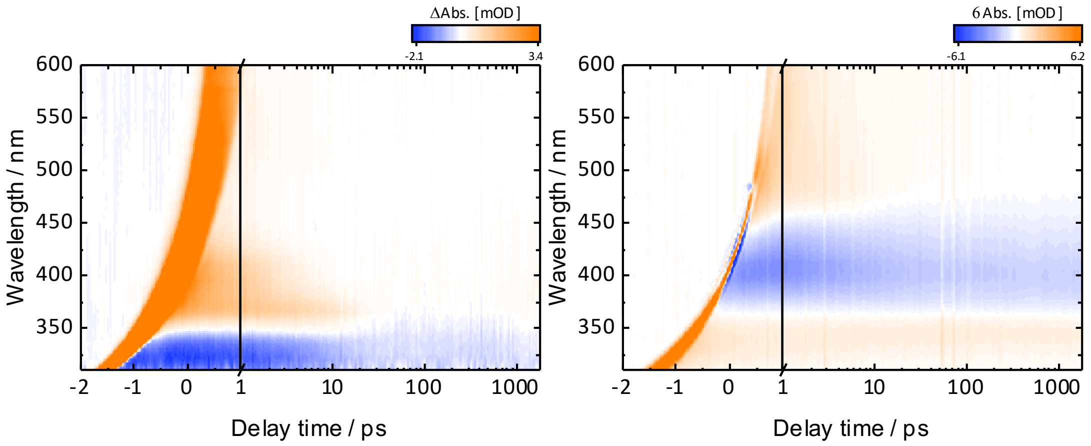

Figure S9: Time-resolved data without chirp correction from the probe light. Left: AB **9** in DMSO excited by 305 nm pulses. Right: MeO-ThpAB **10** in DMSO excited by 400 nm pulses.

All the equivalent transient data in DMSO can be found in Figure S10-S16. Even though the time-resolved data is absence for 0.5 ps of the measurement with 305 nm excitation, all the lifetime analysis shows great resemblance to the measurement in acetonitrile.

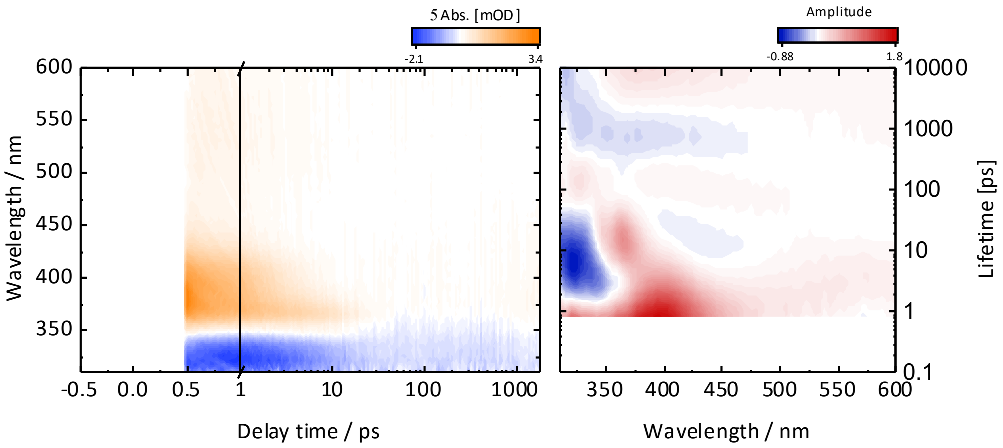

Figure S10: Time-resolved transient data (left) and the lifetime density map (right) of AB **9**, excited by 305 nm pulses.

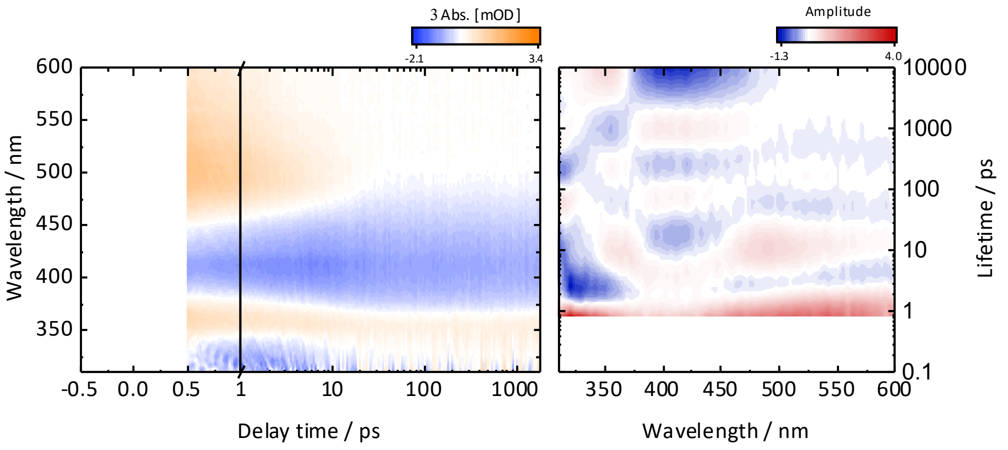

Figure S11: Time-resolved transient data (left) and the lifetime density map (right) of MeO-TphAB-AB **4**, excited by 305 nm pulses.

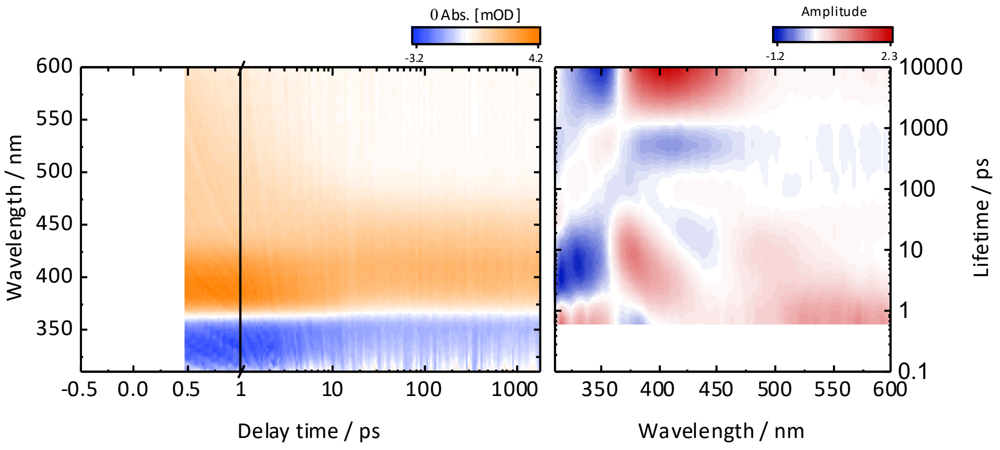

Figure S12: Time-resolved transient data (left) and the lifetime density (right) map of MeO-TphAB-AB **4**, excited by 305 nm pulses, while being illuminated by 420 nm LED (PSS 420 nm).

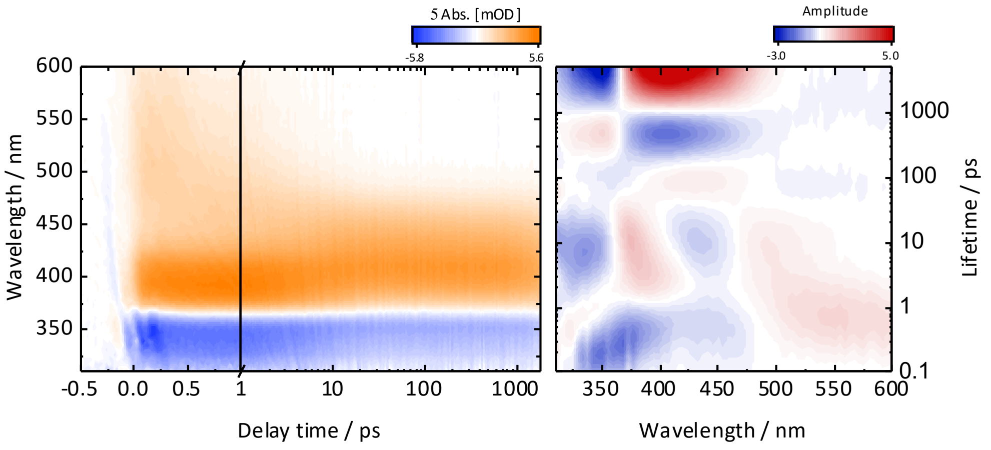

Figure S13: Time-resolved transient data (left) and the lifetime density (right) map of MeO-TphAB-AB **4**, excited by 340 nm pulses, while being illuminated by 420 nm LED (PSS 420 nm).

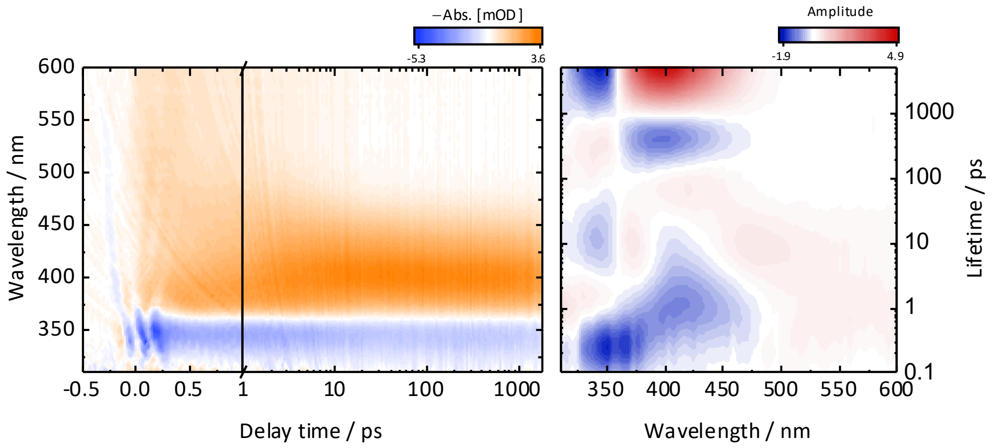

Figure S14: Time-resolved transient data (left) and the lifetime density (right) map of MeO-TphAB **10**, excited by 340 nm pulses, while being illuminated by 420 nm LED (PSS 420 nm).

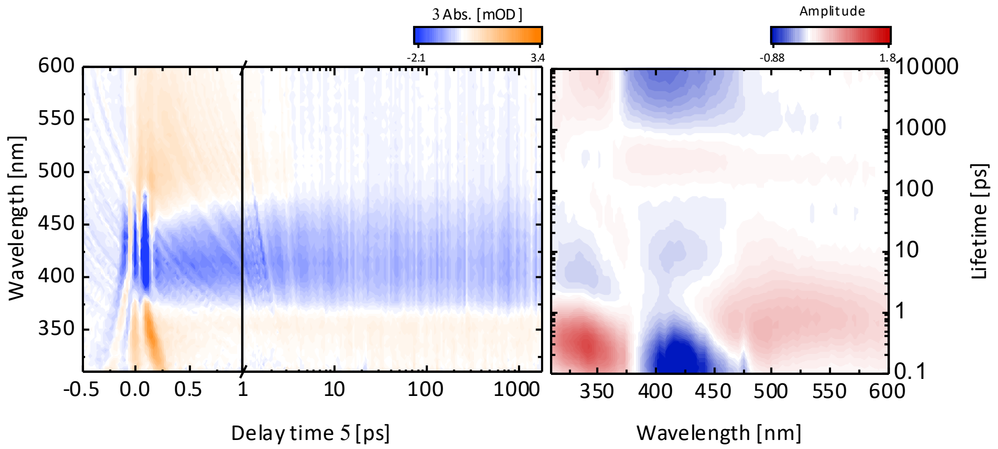

Figure S15: Time-resolved transient data (left) and the lifetime density (right) map of MeO-TphAB-AB **4**, excited by 420 nm pulses.

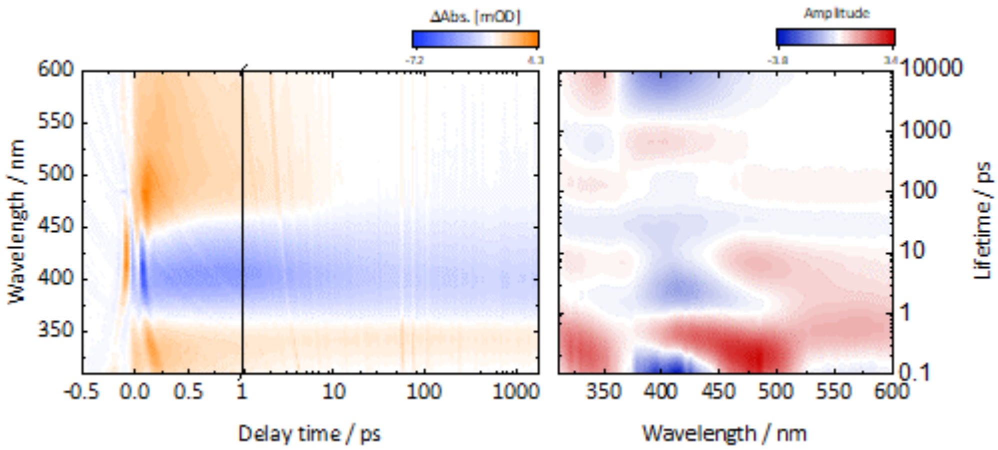

Figure S16: Time-resolved transient data (left) and the lifetime density (right) map of MeO-TphAB **10**, excited by 400 nm pulses.

References

[27] C. Feldmeier, H. Bartling, E. Riedle, R. M. Gschwind, *J. Magn. Reson.* **2013**, *232*, 39.

[28] R. R. Ernst, W. A. Anderson, *Rev. Sci. Instr.* **1966**, *37*, 93.

[29] a) A. H. Heindl, H. A. Wegner, *Chem. Eur. J.* **2020**, *26*, 13730; b) A. H. Heindl, H. A. Wegner, *Beilstein J. Org. Chem.* **2020**, *16*, 22.

[30] MATLAB version 2024b Update 3 (24.2.0.2806996), The MathWorks Inc. (Natick, Massachusetts, United States, 2024.

[31] Y. Shao, et al., *Mol. Phys.* **2015**, *113*, 184–215.

[32] F. Neese, *Wiley Interdiscip. Rev. Comput. Mol. Sci.* **2012**, *2*, 73–78.

[33] T. Yanai, D. P. Tew, N. C. Handy, *Chem. Phys. Lett.* **2004**, *393*, 51–57.

[34] F. Weigend, R. Ahlrichs, *Phys. Chem. Chem. Phys.* **2005**, *7*, 3297–3305.

[35] a) S. Grimme, J. Antony, S. Ehrlich, H. Krieg, *J. Chem. Phys.* **2010**, *132*, 154104; b) S. Grimme, S. Ehrlich, L. Goerigk, *J. Comput. Chem.* **2011**, *32*, 1456–1465.
